# Genome-wide association study of human papillomavirus infection and persistence in women living with HIV in Rwanda

**DOI:** 10.1186/s13027-026-00815-x

**Published:** 2026-10-26

**Authors:** Åsa Torinsson Naluai, Schifra Uwamungu, Maria Andersson, Bethelehem Nigussie Zewdie, Claude Mambo Muvunyi, Emile Bienvenu, Bengt Hasséus, Daniel Giglio

**Affiliations:** 1 Department of Laboratory Medicine, Institute of Biomedicine and Core Facilities, Sahlgrenska Academy, University of Gothenburg, Gothenburg, Sweden; 2 Department of Pharmacology, Institute of Neuroscience and Physiology, Sahlgrenska Academy, University of Gothenburg, Gothenburg, Sweden; 3 Department of Biomedical Laboratory Sciences, School of Health Sciences, College of Medicine and Health Sciences, University of Rwanda, Kigali, Rwanda; 4 Department of Infectious Diseases, Institute of Biomedicine, Sahlgrenska Academy, University of Gothenburg, Gothenburg, Sweden; 5 Communicable and Non-communicable Diseases Research Directorate, Armauer Hansen Research Institute, Addis Ababa, P.O. Box 1005, Ethiopia; 6 Department of Clinical Biology, School of Medicine and Pharmacy, College of Medicine and Health Sciences, University of Rwanda, Kigali, Rwanda; 7 Rwanda Biomedical Centre, Kigali, Rwanda; 8 Rwanda Food and Drugs Authority, Kigali, Rwanda; 9 Department of Pharmacy, School of Medicine and Pharmacy, University of Rwanda, Kigali, Rwanda; 10 Department of Oral Medicine and Pathology, Institute of Odontology, Sahlgrenska Academy, University of Gothenburg, Gothenburg, Sweden; 11 Department of Medicine and Oncology, Southern Älvsborg Hospital, Borås, Sweden; 12 Department of Oncology, Institute of Clinical Sciences, Sahlgrenska Academy, University of Gothenburg, Blå Stråket 2, Gothenburg, SE-41345 Sweden

**Keywords:** Human papillomavirus, HIV, GWAS, Single nucleotide polymorphism, Cervical cancer, Africa

## Abstract

**Background:**

We conducted an explorative genome-wide association study (GWAS) in East Africa to identify host genetic variants associated with the acquisition and persistence of human papillomavirus (HPV) infection.

**Methods:**

We genotyped 385 Rwandan women living with HIV and selected host biomarker assay signals from genital swab samples using qPCR. We compared women who cleared HPV over a two-year interval (operationally defined as persistent infection), with those who did not. Exploratory gene-based aggregation analyses of rare and low-frequency variants represented on the genotyping array were also performed.

**Results:**

In the primary cluster-stratified Cochran–Mantel–Haenszel analysis, rs13158830 within PPARGC1B exceeded the conventional genome-wide significance threshold for persistent HPV infection. The nearby MIR378A gene-expression assay signal was higher in HPV-positive women but did not distinguish clearance from persistence.

**Conclusions:**

We identified an exploratory host genetic association with persistent HPV infection and several additional candidate loci. The lead rs13158830 signal remained evident in Fisher exact testing and PC-adjusted Firth regression, but the small number of carriers produced substantial uncertainty. These findings require replication in independent cohorts.

**Clinical trial registration:**

Not applicable.

**Supplementary Information:**

The online version contains supplementary material available at https://doi.org/10.1186/s13027-026-00815-x.

## Introduction

Persistent infection with human papillomavirus (HPV) is the most important risk factor for the development of cervical cancer, the most common cause of cancer-related death among sub-Saharan African women [1, 2]. In East Africa, cervical cancer screening is predominantly offered to risk groups, e.g., women living with HIV (WLWH). Still, the number of WLWH offered cervical cancer screening is low. We showed that 55% of WLWH attending the HIV clinic at the University Teaching Hospital of Kigali (CHUK), Rwanda, have previously undergone a Pap smear test [3]; the number was 28% among women attending the HIV clinic at the Black Lion Hospital in Addis Ababa, Ethiopia [4]. Rwanda was the first country in Africa to vaccinate schoolgirls, and cervical cancer screening program employing HPV screening has also been initiated [5, 6].

At present, it is not known how to screen out HPV infections that will become persistent [7]. In the era of effective antiretroviral treatment (ART), WLWH have a six times higher risk of developing cervical cancer compared to HIV-negative women [8, 9]. Compounding the problem, girls living with HIV have a weaker immunological response to HPV vaccinations [10]. We previously showed that 56% of HPV infections in WLWH in Rwanda were persistent (> 2 years persistence) [7]. Using proteomics, we identified a panel of cervical biomarkers (LIMCH1, SGTA, BLOC1S5, EIF1, GRPEL1, OPTN, and EZR) that correlated with the risk of developing persistent cervical HPV infections [11].

Genome‑wide association studies (GWAS) have established that human leukocyte antigen (HLA) genes influence the risk of cervical cancer [12]. Yet few genomic studies have examined the earlier steps in the disease continuum, specifically, why some women develop persistent high‑risk HPV infections. Only two GWAS from the African continent (both from Nigeria) have investigated host genetic susceptibility to HPV‑related disease [13, 14], and no previous study has contrasted women with persistent HPV infection against those who cleared their infection.

In this GWAS, we assessed whether host genetic variation influences susceptibility to cervical HPV infection and development of persistent HPV infections among WLWH in Rwanda. We also examined the expression of genes identified in the GWAS and validated protein biomarkers previously reported in an independent cohort of Rwandan WLWH [11].

## Methods

### Cohort

The cohort has been presented previously [3, 15]. WLWH (*N* = 463) receiving ART at CHUK, Kigali, Rwanda, were recruited to the study in 2021. Eligibility criteria included: ≥21 years of age, HIV diagnosis, and voluntarily seeking HPV screening. Exclusion criteria at inclusion included: prior HPV vaccination, a present or previous diagnosis of cervical lesions/cancer, visible or known vaginal or cervical infections (besides HPV), lack of sexual activity with others, or any condition that might prevent compliance with the study protocol.

Participants completed an interview (see questionnaire; Supplementary file 1), after which a physician collected vaginal and cervical swab samples for HPV testing using the Aptima Multitest Swab (Hologic Inc., Marlborough, MA, USA). Additional swabs from the vagina and cervix were placed in RIPA buffer (Thermo Fisher, Rockford, Illinois, USA) for biomarker testing. At baseline, blood samples were collected from participants into EDTA tubes for GWAS analysis (see below) and subsequently stored at − 70 °C. Samples were diluted 1:2 in phosphate buffered saline and nucleic acid from 200 µl specimen was extracted by a MagNA Pure LC instrument (Roche Molecular, Mannheim, Germany) using the DNA Isolation I kit. The nucleic acids were eluted in 100 µl volume and DNA concentration was quantified using a Qubit 4 Fluorometer (Invitrogen, Thermo Fisher Scientific, Waltham, MA, USA).

Pap smear samples were collected with a wooden spatula and cyto-brush. At the 2-year follow-up in 2023, 385 women were interviewed again (see questionnaire; Supplementary file 2) and underwent the same procedures as above (Figures S1-S2). BioRender was used to create the study layout illustration.

### Real-time quantitative PCR and cytology

Real-time quantitative PCR (qPCR) was conducted to detect 12 h-HPV types (HPV16, 18, 31, 33, 35, 39, 45, 51, 52, 56, 58, and 59), two low-risk types (HPV6 and 11), and beta-globin (housekeeping gene), as previously described (see Supplementary methods and Table S1) [3, 15]. qPCR was also performed for selected host biomarkers near the chromosome region 5q lead locus (for TaqMan probes see Table S1). The MIR378A measurement used the Thermo Fisher Scientific TaqMan Gene Expression Assay Hs04273260_s1; this is not a mature-miRNA-specific assay. For biomarker assays, cDNA was prepared directly from RIPA-stored cervical lysate using the High-Capacity cDNA Reverse Transcription Kit as detailed in the Supplementary Methods. DNase treatment and -RT controls were not included; therefore, genomic-DNA contribution to the Hs04273260_s1 signal cannot be excluded. Cytological samples were classified in accordance with the 2014 Bethesda System (see Supplementary methods for further details).

### Outcomes with respect to HPV infection and persistence

Women were classified as ever HPV-infected if they tested positive for any HPV type at baseline and/or follow-up, and never infected if HPV-negative at both visits. HPV-negative women at baseline who were lost to follow-up were excluded. For the persistence analyses, women who were HPV-positive in the cervix and/or vagina at baseline and had cleared all baseline infections at follow-up were classified as cleared. Women with the same HPV type detected at baseline and follow-up were operationally classified as having persistent infection; women retaining all baseline infections were classified as fully persistent, whereas women retaining some but not all baseline infections were classified as partially persistent. The cleared group was compared either with the fully persistent group or with the combined partially/fully persistent group. Women who were HPV-negative at baseline but HPV-positive at follow-up were excluded from the persistent analyses. Since HPV status was measured at only two visits approximately two years apart, same-type redetection cannot distinguish uninterrupted persistence from clearance followed by reinfection or intermittent detectability.

### GWAS

Illumina´s Global Screening Array Multiple Disease (GSA-MD; 730 059 SNPs) was performed on DNA extracted from baseline blood samples [16, 17]. The primary genome-wide association analyses used cluster-stratified Cochran–Mantel–Haenszel (CMH) tests in PLINK (v1.9 and v2.0) [18]. Principal component analysis (PCA), Manhattan plots, genomic inflation factors (λGC), and Q–Q plots were used to evaluate population structure and genome-wide calibration (Figures S3-S4; see Supplementary Methods). Gene-based SKAT/SKAT-O analyses were restricted to rare and low-frequency variants represented on the genotyping array and were considered exploratory [19], For the lead persistence variant rs13158830, we additionally performed a two-sided Fisher exact test of carrier status and Firth penalized logistic regression adjusted for PC1-PC4 as sensitivity analyses.

### Pathway analysis

For exploratory biological annotation, genes located within 100 kb upstream or downstream of each of the top 500 SNPs from the three GWAS comparisons were identified using a fixed physical window and evaluated with Enrichr. This 200-kb total span was used as a pragmatic proximity-based annotation because a population-matched Rwandan LD reference was not established for this cohort. Physical proximity was not interpreted as evidence that a gene was causal or in linkage disequilibrium with the associated variant. The Enrichr results were treated as hypothesis-generating rather than as formal GWAS gene-set evidence. The modus to identify biological pathways has been applied previously by the research group in celiac disease [20].

### Statistics for gene expression of associated genes

Comparisons between two groups and multiple groups were performed with the Mann-Whitney test and the Kruskal-Wallis test followed by Dunn´s multiple comparisons test, respectively. P-values less than 0.05 were considered significant. IBM SPSS Statistics version 26 (IBM Corp., Armonk, NY, USA) and GraphPad Prism 9.1.0 (GraphPad Software, Inc., San Diego, USA) were used to analyze the data.

## Results

### Prevalence of persistent HPV infections

The demographic characteristics at baseline are displayed in Table 1. Among HPV infected at baseline, 38.6% had more than one HPV infection. The most common type in multiple infections was HPV52 occurring in 35.4% of all multiple infections followed by HPV58 (30.8%), HPV56 (27.7%) and HPV16 (24.6%). One out of two women were HPV-negative at both baseline and follow-up (Table S2); 28.8% of women fully cleared their HPV infections, while an additional 8.1% partially cleared their HPV infections. Persistent HPV infections occurred in 6.2% of women; 6.8% were negative at baseline but had acquired infections with one or more HPV types detected at follow-up (Table S2). Among women who were HPV-positive at baseline, 55 of 166 (33.1%) had persistent HPV infections at follow-up. Among women infected at baseline, 38.6% had multiple HPV infections. The most common HPV types involved in multiple infections were HPV52 (35.4%), HPV58 (30.8%), HPV56 (27.7%), and HPV16 (24.6%).

**Table 1:** Demographic characteristics of the cohort at baseline

Age at baseline	*N*	%
≤ 35	42	10.9
36–45	130	33.8
46–55	149	38.7
≥ 56	64	16.6
**Education level**		
Below secondary school	341	88.6
Complete secondary school	30	7.8
Above secondary school	14	3.6
**Occupation**		
Farming	32	8.3
Civil servant	3	0.8
Business	73	19.0
Sex worker	106	27.5
Unemployed	120	31.2
Others	51	13.2
**CD4 (cell/µL) at baseline**		
< 200	32	8.3
200–500	167	43.4
> 500	184	47.8
Not done	2	0.5
**Viral load (copies) at baseline**		
< 400	371	96.4
400–5000	11	2.9
> 5000	3	0.8
Serious disease*		
No	347	90.1
Cardiovascular disease	13	3.4
Cancer	5	1.3
Hepatitis	8	2.1
Tuberculosis	5	1.3
Rheumatic disease	2	0.5
Renal failure	1	0.3
Unspecified serious disease	5	1.3
**Smoking habit**		
No	330	85.7
Yes	13	3.4
Ex-smoker	42	10.9
**Alcohol consumption habit**		
Never/seldom	239	62.1
Once a month	47	12.2
Once a week	38	9.9
Several times a week	61	15.8
**Marital status**		
Married	75	19.5
Has a partner but not married	52	13.5
Separated/divorced/widowed	159	41.3
Single	99	25.7
**Live birth at baseline**		
0–1	81	21.0
2 and above	304	79.0
**Abortions at baseline**		
0–1	325	84.4
> 1	60	15.6
**Age at first intercourse**		
< 18	214	55.6
≥ 18	171	44.4
**Number of previous sex partners at baseline**		
2–4 partners	243	63.1
> 4 partners	142	36.9
**Ever had gonorrhoea**		
No	266	69.1
Yes	117	30.4
I don´t know	2	0.5
**Ever had syphilis**		
No	295	76.6
Yes	85	22.1
I don´t know	5	1.3
**Ever had chlamydia**		
No	231	60.0
Yes	14	3.6
I don´t know	140	36.4
**Contraceptive method at baseline**		
Male condom	29	7.5
Other contraceptives	121	31.4
Not using contraceptives	235	61.0

Footnote. *Serious disease, i.e., cardiovascular disease, cancer, hepatitis, tuberculosis, rheumatic disease, renal failure and unspecified serious disease. Some participants had two serious diseases

The final numbers of individuals included in the GWAS comparisons were 192 versus 193 (ever infected versus never infected), 24 versus 111 (persistent infection versus full clearance), and 55 versus 111 (persistent/partially persistent infection versus full clearance).

The type-specific HPV persistence was 21.5% (100 of 465 HPV infections; Table S3). Persistent cervical infections with HPV16 and HPV18 occurred in 38.7% and 33.3% of cases, respectively, compared with 16.0% for other HR-HPV types (*p* = 0.0075). Similarly, persistent vaginal infections with HPV16 and HPV18 occurred in 40.7% and 23.1% of cases, respectively, compared with 18.1% for other HR-HPV types (*p* = 0.0326; Table S4). HPV16, HPV35 and HPV58 constituted the most prevalent persistent cervical HPV infections and HPV16, HPV51, HPV35 and HPV6 were the most prevalent persistent vaginal HPV infections (Table S3).

### GWAS for acquiring HPV infections

The top SNPs associated with HPV infection are shown in Table 2A (ever infected versus never infected; top 1000 SNPs in Table S5). The positions of the top 30 SNPs and genes assigned by physical proximity, and the proximity-annotated genes from the 500 SNPs used for exploratory Enrichr analysis, are displayed in Table S6 and Table S7, respectively.

**Table 2:** The top ten single nucleotide polymorphisms (SNPs) associated for each GWAS comparison

**A) The top ten SNPs associated with acquiring HPV.**											
**CHR**	**SNP**	**BP**	**A1**	**FA**	**FUnA**	**P**	**GC**	**FDR BH**	**OR**	**Consequence**	**Genes (Nearest gene in bold)**
9	rs7024949	29,198,980	A	0.37	0.52	7.82E-06	1.61E-05	0.89	0.52	NC transcript variant	**LINGO2**
16	rs3087620	10,623,332	C	0.12	0.22	8.27E-06	1.70E-05	0.89	0.41	*3 prime UTR variant*	ATF7IP2; LINC01290; **EMP2**; TEKT5
21	rs1297265	16,817,051	G	0.49	0.33	8.43E-06	1.73E-05	0.89	1.95	Upstream variant	**AJ006998.2**
23	rs1206209	136,559,899	T	0.33	0.49	8.46E-06	1.74E-05	0.89	0.52	NC transcript variant	**LINC02931**; RAC1P4; ZIC3
15	rs8042868	43,939,642	T	0.34	0.20	1.15E-05	2.31E-05	0.89	2.11	*Missense variant*	TP53BP1; MAP1A; PPIP5K1; CKMT1B; **STRC**;** CATSPER2**; PPIP5K1P1-CATSPER2; CKMT1A; STRCP1; CATSPER2P1; PDIA3; ELL3; SERF2; MIR1282; SERINC4; HYPK; MFAP1; WDR76
4	rs4146476	66,716,573	A	0.40	0.55	1.22E-05	2.45E-05	0.89	0.53	Intergenic variant	EPHA5; **EPHA5-AS1**
12	rs11837546	96,614,816	T	0.36	0.51	1.37E-05	2.72E-05	0.89	0.52	NC transcript variant	LTA4H; LINC02452; **ELK3**; CDK17
8	rs7827867	6,136,512	G	0.28	0.16	3.36E-05	6.29E-05	0.97	2.10	Intergenic variant	MCPH1-DT; **MCPH1**
3	rs7620372	110,908,395	C	0.37	0.52	3.38E-05	6.32E-05	0.97	0.55	Upstream variant	NECTIN3-AS1; **NECTIN3**
1	rs1011903	94,143,719	C	0.29	0.42	4.31E-05	7.94E-05	0.97	0.53	NC transcript variant	FNBP1L; **BCAR3**; BCAR3-AS1; MIR760; DNTTIP2
**B) The top ten SNPs associated with developing persistent HPV infections.**											
**CHR**	**SNP**	**BP**	**A1**	**FA**	**FUnA**	**P**	**GC**	**FDR BH**	**OR**	**Consequence**	**Genes (Nearest gene in bold)**
5	rs13158830	149,172,257	A	0.10	0.00	7.82E-10	9.11E-10	0.00	-	NC transcript variant	*ARHGEF37;* ***PPARGC1B***; *MIR378A; PDE6A; MFFP2; SLC26A2; TIGD6; HMGXB3*
5	rs10463291	149,202,269	A	0.08	0.00	4.73E-08	5.34E-08	0.01	-	NC transcript variant	*ARHGEF37;* ***PPARGC1B***; *MIR378A; PDE6A; MFFP2; SLC26A2; TIGD6; HMGXB3*
3	rs138078234	16,358,552	C	0.04	0.00	2.04E-07	2.27E-07	0.03	-	*Missense variant*	*GALNT15; DPH3; * ***OXNAD1*** *;* *** RFTN1***
9	rs12683783	86,870,808	C	0.21	0.02	7.47E-07	8.25E-07	0.07	14.1	Intergenic variant	***SLC28A3***; *SLC28A3-AS1*
2	rs10170959	221,532,215	C	0.08	0.00	9.63E-07	1.06E-06	0.07	-	NC transcript variant	***ENSG00000288902/AC067956.1***
8	rs7840807	126,968,513	T	0.33	0.07	9.80E-07	1.08E-06	0.07	6.85	Upstream variant	***LINC00861***; ***SOD1P3***
7	rs73191136	108,634,920	T	0.08	0.00	1.60E-06	1.76E-06	0.08	-	NC transcript variant	*LINC02903;* ***BUB3P1***
12	rs77799129	127,996,955	C	0.08	0.00	1.63E-06	1.79E-06	0.08	-	NC transcript variant	*LINC02375;* ***RP11-483I24.4/RP11-35O2.1***; *LINC02411*
9	rs10981622	115,817,156	G	0.13	0.01	1.80E-06	1.98E-06	0.08	28.6	NC transcript variant	*SNX30; SLC46A2; ZNF883;* ***ZFP37***; *FAM225B; FAM225A; SLC31A2; FKBP15; SLC31A1*
4	rs116205824	7,062,086	T	0.04	0.00	1.93E-06	2.11E-06	0.08	-	*3 prime UTR variant*	*KIAA0232; TBC1D14; CCDC96; TADA2B;* ***GRPEL1***; *LINC02447; SORCS2*
**C) The top ten SNPs associated with developing partially/fully persistent HPV infections.**											
**CHR**	**SNP**	**BP**	**A1**	**FA**	**FUnA**	**P**	**GC**	**FDR BH**	**OR**	**Consequence**	**Genes (Nearest gene in bold)**
2	rs2591157	155,648,366	C	0.23	0.06	6.09E-07	1.28E-06	0.27	5.71	NC transcript variant	**KCNJ3**
8	rs7840807	126,968,513	T	0.26	0.07	1.69E-06	3.36E-06	0.27	5.18	Upstream variant	***LINC00861***; ***SOD1P3***
9	rs12683783	86,870,808	C	0.15	0.02	1.86E-06	3.68E-06	0.27	11.58	Intergenic variant	**SLC28A3**; SLC28A3-AS1
23	rs56322118	144,901,400	A	0.29	0.08	2.90E-06	5.59E-06	0.32	4.67	NC transcript variant	**SLITRK2**; MIR892C; MIR890; MIR888; MIR892A; MIR892B; MIR891B
22	rs5992749	18,076,523	A	0.28	0.08	8.23E-06	1.50E-05	0.72	4.20	NC transcript variant	CECR2; **SLC25A18**; **ATP6V1E1;** BCL2L13; BID; MIR3198-1; LINC00528; MICAL3
11	rs16921911	90,249,755	G	0.16	0.02	1.00E-05	1.80E-05	0.74	8.09	NC transcript variant	**DISC1FP1**; MIR4490
3	rs13324022	168,874,071	A	0.10	0.32	2.09E-05	3.61E-05	0.94	0.23	NC transcript variant	LINC01997; **MECOM**
19	rs12984733	10,322,595	T	0.15	0.04	2.17E-05	3.74E-05	0.94	6.97	NC transcript variant	RDH8; MIR5589; C3P1; SHFL; ANGPTL6; PPAN; PPAN-P2RY11; SNORD105; SNORD105B; P2RY11; EIF3G; **DNMT1**; S1PR2; MIR4322; MRPL4; LIMASI; ICAM1; ICAM4-AS1; ICAM4; ICAM5; ZGLP1; FDX2-ZGLP1; FDX2; RAVER1; ICAM3; TYK2; CDC37; MIR1181
23	rs3810704	144,904,252	A	0.24	0.07	2.35E-05	4.04E-05	0.94	4.34	*5 prime UTR variant*	**SLITRK2**; MIR892C; MIR890; MIR888; MIR892A; MIR892B; MIR891B
11	rs7120512	84,383,445	A	0.38	0.63	2.39E-05	4.11E-05	0.94	0.36	*NMD variant*	**DLG2**

Footnote. CHR=Chromosome number of the tested SNP; SNP = Single Nucleotide Polymorphism; BP=Base Pair; A1 = Effect allele (the allele tested for association); FA=Allele frequency in affected; FUnA=Allele frequency in unaffected; P = The unadjusted p-value from the Mantel-Haenszel test before multiple testing correction; GC=Genomic Control adjusted p-value (corrects for population stratification via inflation factor); FDR BH=False Discovery Rate (FDR) adjustment by Benjamini-Hochberg (BH) method; OR=Odds Ratio for the effect allele (A1); Consequence=Calculated variant consequences in Ensembl Variant Effect Predictor (VEP); NC = Non Coding; UTR=UnTranslated Region; NMD=Nonsense-mediated mRNA decay

**Fig. 1:**
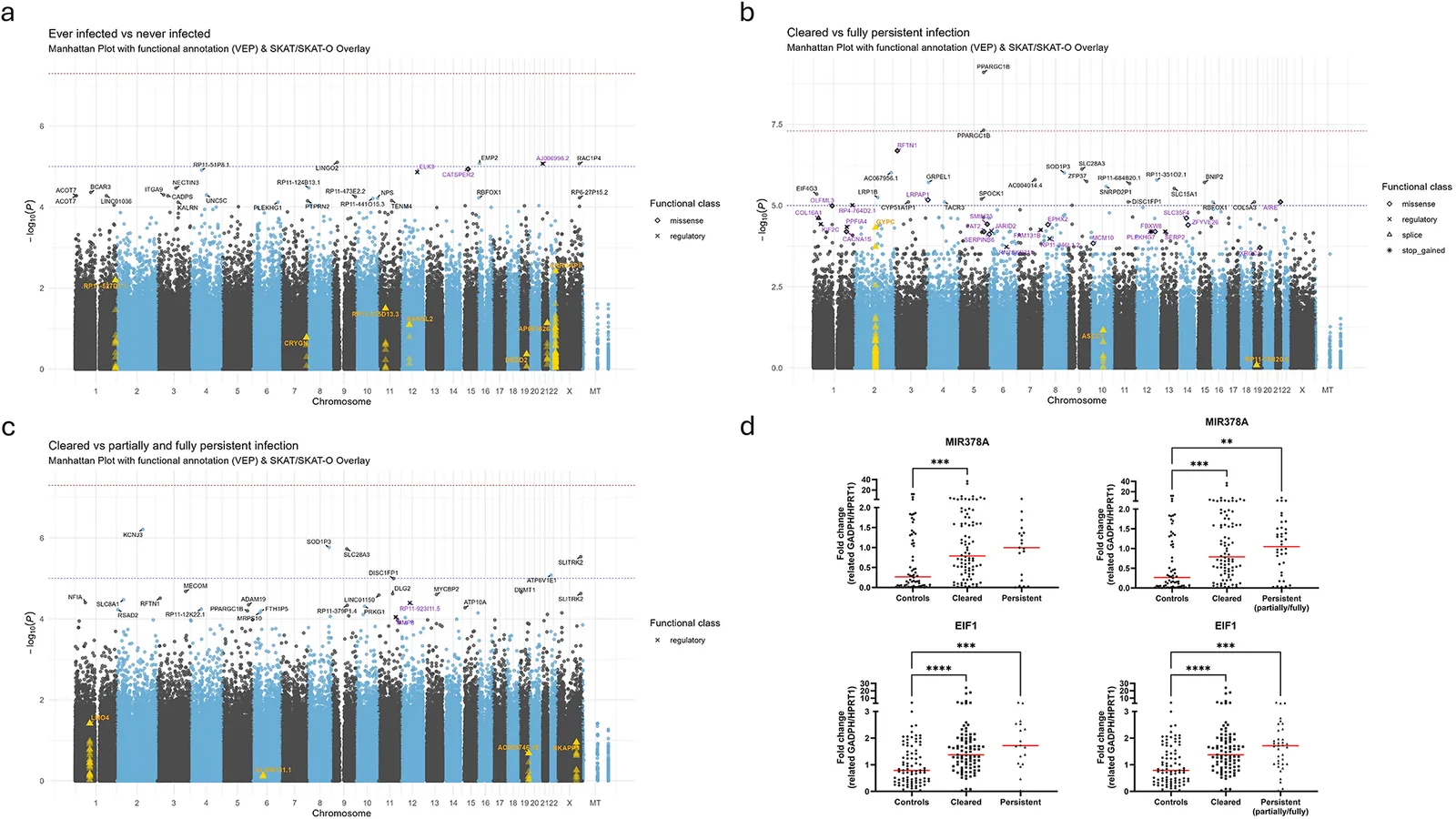
Manhattan plot of the overall associations with (a) acquiring HPV, (b) developing persistent HPV infections and (c) developing partially/fully persistent HPV infections. The red line indicates the genome-wide levels of significance threshold and the blue line indicates the threshold for suggestive genome-wide associations. Top genes identified by the Sequence Kernel Association Test (SKAT) are highlighted in yellow, while top genes based on functional annotations from the Variant Effect Predictor (VEP) are highlighted in purple. (d) Expression of MIR378A and EIF1 mRNAs in different outcome groups. Controls (HPV negative at baseline and follow-up; *n* = 193), fully HPV cleared (*n* = 111), partially HPV persistent (*n* = 31) and fully HPV persistent (*n* = 24). The red lines indicate median values. **<0.01, ***<0.001 and ****<0.0001

A Manhattan plot of the overall associations is shown in Fig. 1a. Exploratory literature annotation identified several genes near the top SNPs relevant to HPV and/or HPV-related cancer for example *EMP2* (rs3087620 in exon 5), *TP53BP1* and *PDIA3* (rs8042868 in *CATSPER2* exon 3), *RBFOX1* (rs12934429 in *RBFOX1* intron 1), *PTPRN2* (rs4716515 in intron 11) and *KALRN* (rs1444768 in intron 1; see supplementary gene and reference list). Exploratory gene-based aggregation testing of rare and low-frequency variants represented on the array highlighted the Rho‑GTPase–regulating gene *ARHGAP8*.

Exploratory Enrichr annotation of genes assigned to the top 500 SNPs yielded terms including COVID-19 (Hospitalized Vs Not Hospitalized) and Inflammatory Skin Disease (Table 3; full list in Table S8). Among the genes associated with the top 30 biological pathways, we identified *HPSE2* (rs2494657), *KALRN* (rs1444768) and *SGCZ* (rs17655231), with a connection to COVID-19 (hospitalized vs. not hospitalized), and *PTPRN2* (rs4716515), with a connection to Inflammatory Skin Disease.

**Table 3:** Top exploratory Enrichr terms from proximity-annotated genes for the HPV infection GWAS

Term	Overlap	*P*-value	Adjusted *P*-value	Odds Ratio	Combined Score	Genes	Chromosome
HR-HER2- Amplification-19P13 2 ERBB2 (PMID: 28027327)	29/65	2.01E-16	6.68E-13	10.32	372.8	DOCK6; KANK2; SWSAP1; ODAD3; PLPPR2; PRKCSH; SLC44A2; RAB3D; KEAP1; KRI1; ELAVL3; TMEM205; PDE4A; S1PR5; LDLR; AP1M2; CDKN2D; CCDC159; EPOR; DNM2; QTRT1; TSPAN16; ILF3; CDC37; ANGPTL8; RGL3; ZNF653; SPC24; ATG4D	19
HR HER2- Deletion-19P13 2 ERBB2 (PMID: 28027327)	29/74	1.31E-14	2.17E-11	8.25	263.7	DOCK6; KANK2; SWSAP1; ODAD3; PLPPR2; PRKCSH; SLC44A2; RAB3D; KEAP1; KRI1; ELAVL3; TMEM205; PDE4A; S1PR5; LDLR; AP1M2; CDKN2D; CCDC159; EPOR; DNM2; QTRT1; TSPAN16; ILF3; CDC37; ANGPTL8; RGL3; ZNF653; SPC24; ATG4D	19
RNA Nuclease Activity (GO:0004540)	16/70	3.81E-05	2.53E-02	3.76	38.2	TSNAX; ENDOG; RNASE11; RNASET2; RNASE6; RNASE12; RNASE2; RNASE1; SND1; SMG6; ENDOU; PPP1R8; ANG; DCPS; EXOSC2; ANKZF1	1, 2, 6, 7, 9, 11, 12, 14, 17
Paraxanthine Levels In Elite Athletes	5/6	1.21E-05	2.87E-02	63.1	714.3	CYP2A6; CAMK1D; NOX5; BMPR1B; ABCG2	4, 10, 15, 19
COVID-19 (Hospitalized Vs Not Hospitalized)	17/74	2-05E-05	2.87E-02	3.78	40.8	HPSE2; VDR; LINC01250; SFTPD; ACTN4; KALRN; SGCZ; TIAM2; NFASC; ADGRB3; SSPN; PIEZO1; DMD; MALRD1; CTNNA2; ATP13A3; LINC00378	1, 2, 3, 6, 8, 10, 12, 13, 16, 19, X
HR-HER2- Deletion-5Q33 3 ERBB2 (PMID: 28027327)	9/24	2.95E-05	3.26E-02	7.59	79.2	FAM200C; TENM2; C1QTNF2; PTTG1; FABP6; WWC1; ATP10B; SLU7; CCNJL	5
BDP1	3/4	1.51E-03	4.67E-02	37.8	245.7	ABL1; FYN; ARHGAP35	6, 9, 19
Conjunctival UV Autofluorescence (CUVAF)	4/5	1.38E-04	7.74E-02	50.4	448.4	LINC02354; FKRP; SLC1A5; HDAC7	12, 19
Diamine Acetyltransferase 2 Levels	4/5	1.38E-04	7.74E-02	50.4	448.4	FXR2; TNFSF12; SAT2; SHBG	17
N6-acetyllysine Levels	4/5	1.38E-04	7.74E-02	50.4	448.4	SOX15; SAT2; SHBG; MPDU1	17
Inflammatory Skin Disease	12/49	1.74E-04	8.11E-02	4.1	35.5	QTRT1; GRM4; PTPRN2; REV3L; ELMO1; PDE4A; LINC02429; PRR5L; SLIT3; TGM3; R3HCC1L; MFSD4B	4, 5, 6, 7, 10, 11, 19

### GWAS for the development of persistent versus clearance of HPV infections

The SNPs most strongly associated with fully persistent HPV infections versus clearance are shown in Table 2B (top 1000 SNPs in Table S9). The positions of the top 30 SNPs and proximity-annotated genes are displayed in Table S10 and the proximity-annotated genes from the top 500 SNPs are displayed in Table S7. A Manhattan plot is shown in Fig. 1b.

In the primary cluster-stratified CMH analysis, rs13158830 on chromosome 5 exceeded the conventional genome-wide significance threshold (*p* = 7.82 × 10^− 10^). All 110 women who cleared HPV were G/G at rs13158830; among 24 women with fully persistent infection, 20 were G/G, three were A/G, and one was A/A. Thus, 4/24 (16.7%) persistent cases carried the A allele versus 0/110 clearers. A two-sided Fisher exact test based on carrier status yielded *p* = 8.28 × 10^− 4^. In a Firth penalized logistic regression adjusted for PC1-PC4, the rs13158830 dosage association remained statistically significant (β = 4.09; OR = 59.6; profile-likelihood 95% CI approximately 5.14–8.38 × 103; *p* = 9.18 × 10^− 5^; *n* = 134). The very wide confidence interval reflects the small number of carriers, and the OR should not be interpreted as a precise effect-size estimate. Genome-wide calibration for this CMH scan was good (λGC = 1.010 across 437 162 valid SNP tests); the Q-Q plot followed the null expectation across most of the distribution with upward deviation in the upper tail (Supplementary Figure S3). Several other top-ranked low-frequency variants co-occurred in the same small number of cases; high inter-variant correlations were therefore interpreted cautiously as possible co-carriage rather than evidence of linkage or epistasis.

We found one genome-wide significant SNP on chromosome 5 (rs13158830). Of the top 30 SNPs, 26 had an allele frequency of < 5% and the majority (20) were non-existent in those first infected, but who cleared their infection, indicating the possible involvement of many rare variants with possibly a stronger effect size influencing persistent infection. Several of the top 30 SNPs located on different chromosomes were highly correlated with one another. (Table 4). The missense variant rs138078234 in *RFTN1* exhibited strong correlation (r > 0.7) with five other top-ranking SNPs. Additionally, the intergenic variant rs62261221 was found to be in perfect correlation (r = 1.0) with rs34219046, a missense variant in *AIRE* exon 5. On chromosome 4, rs116205824, a 3’ UTR variant in *GRPEL1* exon 4, was correlated with four other SNPs (*r* > 0.7): including the *RFTN1* missense variant (*R* = 0.7) and a perfect correlation (*R* = 1.0) with rs11631407, an intergenic variant on chromosome 15, downstream of *BNIP2* and *GTF2A2* (Table 4).

**Table 4:** The correlation between genotypes at two loci (SNP1 & SNP2), using the top 30 SNPs from the analysis comparing persistent versus cleared HPV infection

SNP 1				SNP 2				*R*
ID	**CHR**	**Gene**	**Consequence**^**1**^	**ID**	**CHR**	**Gene**	**Consequence**^**1**^	
rs62261221	3	*CYP51A1P1*	intergenic variant	rs34219046	21	*AIRE*	*missense variant*	1.00
rs116205824	4	*GRPEL1*	*3 prime UTR variant*	rs11631407	15	*BNIP2*	intergenic variant	1.00
rs75357225	1	*EIF4G3*	*NMD transcript variant*	rs201377092	4	*TACR3*	*5 prime UTR variant*	0.81
rs116205824	4	*GRPEL1*	*3 prime UTR variant*	rs72987261	11	*RP11-684B20.1*	NC transcript variant	0.81
rs72987261	11	*RP11-684B20.1*	NC transcript variant	rs11631407	15	*BNIP2*	intergenic variant	0.81
rs13158830	5	*PPARGC1B*	NC transcript variant	rs10463291	5	*PPARGC1B*	downstream gene variant	0.81
rs144740096	1	*OLFML3*	exon variant	rs79417794	2	*LRP1B*	NC transcript variant	0.78
rs201377092	4	*TACR3*	*5 prime UTR variant*	rs10463291	5	*PPARGC1B*	downstream gene variant	0.74
rs138078234	3	*RFTN1*	*missense variant*	rs62261221	3	*CYP51A1P1*	intergenic variant	0.70
rs138078234	3	*RFTN1*	*missense variant*	rs116205824	4	*GRPEL1*	*3 prime UTR variant*	0.70
rs138078234	3	*RFTN1*	*missense variant*	rs201377092	4	*TACR3*	*5 prime UTR variant*	0.70
rs138078234	3	*RFTN1*	*missense variant*	rs11631407	15	*BNIP2*	intergenic variant	0.70
rs138078234	3	*RFTN1*	*missense variant*	rs34219046	21	*AIRE*	*missense variant*	0.70
rs116205824	4	*GRPEL1*	*3 prime UTR variant*	rs117131967	16	*LITAF*	downstream gene variant	0.70
rs77799129	12	*RP11-483I24.4*	NC transcript variant	rs117131967	16	*LITAF*	downstream gene variant	0.70
rs11631407	15	*BNIP2*	intergenic variant	rs117131967	16	*LITAF*	downstream gene variant	0.70

Footnote. Missense variants, those in untranslated regions (UTR) and those altering Nonsense-mediated mRNA decay (NMR) are more likely to (but not necessarily) have a direct effect altering the gene function (underlined). CHR=chromosome; BP=base pair; SNP=single nucleotide polymorphism; R = the Pearson correlation coefficient. NC = Non-Coding, UTR=UnTranslated Region, NMD=Nonsense-mediated mRNA decay, Consequence = Calculated variant consequences in Ensembl Variant Effect Predictor (VEP) [37]

Exploratory literature annotation identified several genes near the top persistence-associated SNPs with prior reports related to HPV or HPV-related cancer, including *PPARGC1B*/*MIR378A*, *RFTN1*, *GRPEL1*, *LRP1B* and *RBFOX1* (see Supplementary gene and reference list).

In the exploratory gene-based aggregation analysis of rare and low-frequency variants represented on the genotyping array, *GYPC* was among the highlighted genes. Exploratory Enrichr annotation of proximity-mapped genes from the top 500 SNPs yielded terms including HIV-1 control; Alpha-L-iduronidase Levels; Asthma; and Vaginal Microbiome MetaCyc Pathway (PWY-5860|superpathway Of Demethylmenaquinol-6 Biosynthesis I; Table 5; full list in Table S11).

**Table 5:** Top exploratory Enrichr terms from proximity-annotated genes for the fully persistent HPV GWAS

Term	Overlap	*P*-value	Adjusted *P*-value	Odds Ratio	Combined Score	Genes	Chromosome
HR-HER2- Deletion-19P13 3 ERBB2 (PMID: 28027327)	33/111	4.59E-11	1.60E-07	4.75	113.1	MIDN; SHC2; DAZAP1; C2CD4C; TPGS1; CBARP; FSTL3; RPS15; STK11; SBNO2; FAM174C; MIER2; BSG; POLR2E; MADCAM1; FGF22; GAMT; GPX4; CIMAP1D; CIRBP; PRSS57; ABCA7; POLRMT; ATP5F1D; ARHGAP45; RNF126; GZMM; CDC34; EFNA2; NDUFS7; PLPP2; SPMAP2; HCN2	19
Elevated Serum Carcinoembryonic Antigen Levels	8/9	2.05E-08	3.13E-05	87.54	1549.5	SULT2B1; SEC1P; CA11; DBP; FAM83E; FUT2; FUT1; ABO	9, 19
Protein FAM3D Levels	8/9	2.05E-08	3.13E-05	87.54	1549.5	MED22; SEC1P; FAM83E; NTN5; FUT2; RPL18; SLC2A6; ABO	9, 19
Intercellular Adhesion Molecule 1 Levels	9/12	3.59E-08	3.64E-05	32.84	563.0	MRPL4; DNMT1; RAVER1; ZGLP1; FDX2; PDE4A; TYK2; S1PR5; ABO	9, 19
HR-HER2- Deletion-2Q37 3 ERBB2 (PMID: 28027327)	26/101	1.33E-07	2.32E-04	3.88	61.4	DTYMK; DGKD; RNPEPL1; TEX44; HJURP; ING5; NEU4; GPC1; D2HGDH; TRPM8; KIF1A; GAL3ST2; ANKMY1; UGT1A6; GPR35; AQP12A; DUSP28; B3GNT7; CAPN10; NCL; ATG4B; ACKR3; NMUR1; USP40; PDCD1; PTMA	2
2Q37 Copy Number Variation Syndrome WP5224	31/135	1.47E-07	9.54E-05	3.34	52.5	MROH2A; RTP5; DTYMK; DGKD; RNPEPL1; HJURP; ILK; GABARAP; ING5; CYTH2; NEU4; GPC1; D2HGDH; TRPM8; MAP4; KIF1A; GAL3ST2; ANKMY1; UGT1A6; MEF2A; MYOG; GPR35; AQP12A; PDX1; DUSP28; RRAGB; CAPN10; ATG4B; ACKR3; USP40; PDCD1	1, 2, 3, 11, 13, 15, 17, 19, X
HIV-1 control	10/19	6.90E-07	7.83E-04	12.35	175.2	DDR1; HCP5; CDSN; NOTCH4; PSORS1C3; RNF39; BTNL2; HLA-A; VARS2; MICA	6
Alpha-L-iduronidase Levels	8/12	8.96E-07	6.82E-04	21.88	304.7	GAK; RNF212; SLC26A1; CTBP1; IDUA; DGKQ; TMEM175; FGFRL1	4
Asthma	38/204	1.84E-06	1.04E-03	2.57	33.9	NPSR1; HCP5; CRB1; NNMT; EQTN; NOTCH4; CELF2; C11ORF71; SLC8A1; TRPS1; ADORA1; BBS9; D2HGDH; TRPM8; CSMD3; MICA; MICB; TNFSF18; PDGFRA; LRRIQ3; IL13; C7ORF25; BTNL2; RANBP6; IGFN1; FBXO11; PEX14; MOB3B; RAD51B; IL4; RAD50; CFHR2; GDNF; IRF1; SARDH; HLADRA; TMEM9; TEK	1, 2, 4, 5, 6, 7, 8, 9, 10, 11, 14, 15
Vaginal Microbiome MetaCyc Pathway (PWY-5860|superpathway Of Demethylmenaquinol-6 Biosynthesis I)	8/14	4.66E-06	2.84E-03	14.59	179.1	NLGN1; DLG2; LINC01924; RFX2; SPOCK1; LINC00305; ACSBG2; LRP1B	2, 3, 5, 11, 18, 19

### GWAS for the development of partially and fully persistent versus clearance of HPV infections

The SNPs most strongly significantly associated with the combined partially/fully persistent phenotype versus clearance are shown in Table 2C (top 1000 SNPs in Table S12). The positions of the top 30 SNPs and proximity-annotated genes are displayed in Table S13, and the proximity-annotated genes from the top 500 SNPs are included in Table S7. A Manhattan plot of the overall associations is shown in Fig. 1c.

Exploratory literature annotation identified several genes near the top SNPs with prior reports relevant to HPV or HPV-related cancer, including *DLG2* and *OXNAD1* (see Supplementary gene and reference list). Exploratory Enrichr annotation of genes assigned to the top 500 SNPs yielded the Vaginal Microbiome MetaCyc Pathway (PWY-5860 super pathway) among the top-ranked terms (Table 6; full list in Table S14).

**Table 6:** Top exploratory Enrichr terms from proximity-annotated genes for the partially/fully persistent HPV GWAS

Term	Overlap	*P*-value	Adjusted *P*-value	Odds Ratio	Combined Score	Genes	Chromosome
HR HER2- Deletion-20P13 ERBB2 (PMID: 28027327)	18/34	2.42E-11	8.30E-08	12.37	302.5	NRSN2; TMEM74B; DEFB127; RAD21L1; SIRPB2; SOX12; DEFB132; SDCBP2; FKBP1A; NSFL1C; C20ORF96; C20ORF202; TBC1D20; SNPH; TRIB3; PSMF1; RBCK1; ZCCHC3	20
HR-HER2- Amplification-19P13 2 ERBB2 (PMID: 28027327)	23/65	1.14E-09	1.96E-06	6.03	124.2	DNMT1; P2RY11; RDH8; PPAN; KEAP1; ICAM4; TYK2; FBXL12; MRPL4; UBL5; OLFM2; RAVER1; ANGPTL6; ZGLP1; CDC37; COL5A3; FDX2; EIF3G; PDE4A; PIN1; S1PR2; S1PR5; SHFL	19
Elevated serum carcinoembryonic antigen levels	9/11	9.70E-09	1.11E-05	49.27	909.0	SULT2B1; CA11; DBP; FAM83E; FUT2; RPL18; FUT1; ABO; FUT3	9, 19
Elevated Serum carcinoembryonic antigen levels	8/9	2.05E-08	3.13E-05	87.54	1549.5	SULT2B1; SEC1P; CA11; DBP; FAM83E; FUT2; FUT1; ABO	9, 19
Protein FAM3D Levels	8/9	2.05E-08	3.13E-05	87.54	1549.5	MED22; SEC1P; FAM83E; NTN5; FUT2; RPL18; SLC2A6; ABO	9, 19
Intercellular Adhesion Molecule 1 Levels	9/12	3.59E-08	3.64E-05	32.84	563.0	MRPL4; DNMT1; RAVER1; ZGLP1; FDX2; PDE4A; TYK2; S1PR5; ABO	9, 19
Alpha-L-iduronidase Levels	8/12	8.96E-07	6.82E-04	21.88	304.7	GAK; RNF212; SLC26A1; CTBP1; IDUA; DGKQ; TMEM175; FGFRL1	4
Vaginal Microbiome MetaCyc Pathway (PWY-5860|superpathway Of Demethylmenaquinol-6 Biosynthesis I)	8/14	4.66E-06	2.84E-03	14.59	179.1	NLGN1; DLG2; LINC01924; RFX2; SPOCK1; LINC00305; ACSBG2; LRP1B	2, 3, 5, 11, 18, 19
Hair color	17/61	7.27E-06	4.17E-03	4.24	50.2	OCA2; CDT1; SLC24A4; TRAPPC2L; SNAI3; CYBA; CBFA2T3; PABPN1L; APRT; GALNS; CTU2; MIR4722; PIEZO1; ZC3H18; MVD; IL17C; RNF166	14, 15, 16
Resting metabolic rate	8/16	1.71E-05	6.53E-03	10.94	120.0	SULT2B1; LINC01085; HSD17B14; FUT2; ACTL9; ADAMTS10; FGF21; BCAT2	4, 19

### Expression of genes associated with persistent cervical and vaginal HPV infections

Expression assays for genes near the lead SNP rs13158830 locus (*ARHGEF37*, *PPARGC1B*, *MIR378A*, *PDE6A*, *SLC26A2*, *TIGD6* and *HMGXB3)* and genes previously associated with persistent HPV infection (*LIMCH1*, *SGTA*, *BLOC1S5*, *EIF1*, *GRPEL1*, *OPTN* and *EZR)* [11], were first assessed in a test group (*N* = 8; see Table S1 for assay information). Only the MIR378A Hs04273260_s1 assay and EIF1 yielded signals that could be evaluated in the larger sample set (*N* = 348). The MIR378A assay signal and EIF1 expression were higher in both the cleared and partially/fully persistent groups than in women who were HPV-negative at both visits (*P* < 0.01 to *P* < 0.0001; Fig. 1d), but neither measurement distinguished persistence from clearance. Because Hs04273260_s1 is a gene-expression assay rather than a mature-miRNA-specific assay, and because DNase treatment and -RT controls were not performed, the MIR378A signal is interpreted cautiously and may include a genomic-DNA contribution.

## Discussion

We show that persistent HPV infections were common among women living with HIV in Rwanda and identified rs13158830 within intron 1 of PPARGC1B as an exploratory lead association in the primary CMH GWAS. The signal exceeded the conventional genome-wide significance threshold in the primary analysis and remained evident in Fisher exact testing and PC-adjusted Firth regression. However, it was based on only four A-allele carriers among 24 persistent cases and no carriers among 110 clearers, resulting in substantial uncertainty around the effect estimate. This is, to our knowledge, the first GWAS directly contrasting persistent genital HPV infection with clearance in women living with HIV in East Africa.

The present GWAS should be regarded as exploratory. Statistical power was limited, particularly for the fully persistent versus clearance comparison, and making low-frequency associations particularly sensitive to sparse counts. Genome-wide calibration was nevertheless good (λGC = 1.010), and the lead rs13158830 signal remained statistically significant in a carrier-based Fisher exact test and Firth regression adjusted for PC1-PC4. These sensitivity analyses support the presence of an association but do not provide a precise estimate of effect size or substitute for independent replication. The remaining loci, array-based rare/low-frequency variant aggregation analyses, and Enrichr results should be regarded as hypothesis-generating.

None of the top 30 SNPs have been previously linked to HPV-related disease, nor do they overlap with top SNPs reported in GWAS on HPV from Nigeria [13, 14]. The primary finding of this study was the genome-wide significant association between persistent HPV infection and rs13158830, located within intron 1 of *PPARGC1B*. *MIR378A* is embedded within the same intron and has biological relevance to cancer and HPV-related cellular models [21, 22]. In this study, however, the Hs04273260_s1 assay signal was increased in HPV-positive women in both the clearance and persistence groups, and therefore did not specifically support the persistence-versus-clearance genetic association. Moreover, Hs04273260_s1 is a gene-expression assay rather than a mature-miRNA-specific assay, and the absence of DNase treatment and -RT controls means that genomic-DNA contribution cannot be excluded. We therefore do not regard this result as functional validation of rs13158830 and interpret it only as an exploratory observation related to HPV status.

In addition, several nearby genes of the top 30 associated SNPs have previously been linked to HPV or cervical cancer. Many of the top-ranked variants were rare (< 1%). However, given the limited sample size, these findings should be interpreted cautiously and require validation in independent cohorts. Although the sample size is modest, the magnitudes of observed effect sizes suggest the possibility of rare or population specific variants contributing substantially to phenotype risk.

Several low-frequency variants among the top persistence-associated SNPs were highly correlated across chromosomes. Because these variants occurred in very few individuals, perfect or near-perfect correlations can arise when variants are co-carried by the same participants and should not be interpreted as evidence of linkage or epistasis. Formal gene-gene interaction analyses and carrier-exclusion sensitivity analyses were not performed, so these correlations are reported descriptively only. Furthermore, untranslated-region or coding annotation alone is insufficient to infer biological function without experimental validation.

The GRPEL1 3′ UTR variant rs116205824 was the tenth most significant SNP associated with the development of HPV infection. We showed previously that the cervical expression of GRPEL1 was higher in women with persistent HPV infections compared with women clearing their HPV infections and GRPEL1 has been suggested to constitute a blood biomarker to discriminate women with HSIL from women with LSIL [11, 23]. The associated 3 prime SNP variant in *GRPEL1* was highly correlated with top associated SNPs in *RFTN1 (missense variant)*,* BLIP2*,* RP11-684B20.1* and *LRP1B*, which is an integration site for HPV16 leading to downregulation of LRP1B in cervical cancer [24, 25].

We found rs7024949 among the topmost significant SNPs associated with acquiring HPV. This SNP lies in intron 1 of *LINGO2* in chromosome 9p21.1. No other gene but *LINGO2* lies in the vicinity of rs7024949. *LINGO2* is expressed in cervical tissue (GTEX release 6) and SNPs in *LINGO2* has been associated with breast cancer development as well as response to MMR vaccination [26–28]. The second most significant SNP associated with acquiring HPV was rs3087620 (exon 5 of *EMP2*), which is overexpressed in several cancers and constitute a suggested blood biomarker for cervical cancer [29]. Near rs3087620 lies *TP53BP1*, which plays an important role in cervical carcinogenesis [30].

The exploratory Enrichr analysis produced several heterogeneous terms, including the Vaginal Microbiome MetaCyc Pathway (PWY-5860|superpathway Of Demethylmenaquinol-6 Biosynthesis I) ranked among the ten most significantly enriched biological pathways associated with HPV persistence. The vaginal microbiome is an important player in HPV infection [31]. In our Enrichr analysis, one of the identified genes of this pathway was Discs Large MAGUK Scaffold Protein 2 (*DLG2*), which is an integration site for HPV [32]. Integration may tentatively alter the vaginal defences favoring HPV persistence. Another gene of the same pathway was the putative tumor suppressor gene *LRP1B* (see above) [33].

The eukaryotic translation initiation factors (EIFs) play an important role in HPV infection and other viral infections and may constitute drug targets [34, 35]. We previously identified EIF1 as a protein biomarker identifying women who develop persistent HPV infections [11]. Among examined biomarkers (*LIMCH1*, *SGTA*, *BLOC1S5*, *GRPEL1*, *OPTN* and *EZR*) we were only able to detect the expression of *EIF1*. At baseline, EIF1 expression was higher in women who were HPV-positive, including women who subsequently cleared their infections and women who subsequently developed persistent infections, than in women who were HPV-negative at both baseline and follow-up. EIF1 expression did, however, not significantly differ between women who subsequently cleared HPV and those with persistent HPV infection. Therefore, the present transcript result should therefore be interpreted as an association with HPV status rather than a persistence-specific biomarker. Expressional differences of EIF1 on the mRNA and protein levels between the HPV status groups could tentatively depend on differences in the regulation on the translational level [11].

The exploratory SKAT/SKAT-O analysis highlighted GYPC and ARHGAP8. Because the GSA array captures only a subset of rare variation, this analysis should be described as gene-based aggregation testing of rare and low-frequency variants represented on the array rather than as a comprehensive rare-variant analysis. Sequencing and replication in a larger cohort would be required to evaluate these gene-level signals more comprehensively.

The HPV persistence rate in the current cohort (33.1%) was lower than the 56% we observed in our previous cohort of women living with HIV from the same clinic [7]. The disparity in persistence rates could reflect differences in the analytical methods used and the lower number of HPV types analyzed in the present study compared to our previous study (qPCR versus Multiplex Luminex system, Bio-Rad Laboratories) [7]. The persistence rate of HPV in the present study was, however, similar to that reported in another study from Rwanda in women living with HIV, 37.2% [36].

A strength of our study is that we were able to conduct the first GWAS on HPV-related disease and the first assessment of genotypes associated with persistent HPV infections (i.e., persisting for > 2 years) in Africa. Important limitations should be emphasized. First, the fully persistent GWAS included only 24 cases and 110 genotyped clearers, and the lead rs13158830 association was based on four carriers; effect-size estimates are therefore imprecise and require independent replication. Second, HPV status was measured at two visits approximately two years apart, so the operational persistence phenotype cannot exclude clearance followed by same-type reinfection or intermittent detectability. Third, the primary CMH GWAS was not adjusted for all clinical and behavioral factors that may influence HPV persistence, leaving the possibility of residual confounding. Fourth, biomarker samples had been stored in RIPA buffer and cDNA was prepared directly from unextracted lysate without DNase treatment or -RT controls. The MIR378A Hs04273260_s1 assay can detect genomic DNA, so the measured signal cannot be attributed unequivocally to RNA and is not considered functional validation of the genetic association. Finally, the study included women living with HIV only, and generalizability to HIV-negative women is unknown.

In conclusion, rs13158830 represents an exploratory candidate association with persistent HPV infection in this cohort; additional loci, array-based gene aggregation results, and pathway annotations are hypothesis-generating and require validation in larger, independent cohorts.

## Supplementary Information

Below is the link to the electronic supplementary material.

Supplementary Material 1: Additional file 1. Questionnaire in Kinyarwanda and English administered to the study participants at baseline.

Supplementary Material 2: Additional file 2. Questionnaire in Kinyarwanda and English administered to the study participants at the 2-year follow-up.

Supplementary Material 3: Additional file 3. Supplementary figures 1-4. Figure S1. Study flow chart. Figure S2. Study design. Figure S3. Q-Q plot for the cleared versus fully persistent HPV CMH GWAS. Figure S4. Q-Q plot for the cleared versus partially/fully persistent HPV CMH GWAS.

Supplementary Material 4: Additional file 4. Supplementary tables 1-16. Table S1. Primer sequences of HPV types and beta-globin (housekeeping gene) and primers used for analysis of biomarkers and housekeeping genes. Table S2. Women with HPV infections. Table S3. Cervical and vaginal type-specific clearance and persistence of HPV and developing new low-risk and high-risk HPV types at follow-up. Table S4. Type-specific clearance and persistence of low-risk and high-risk HPVs. Table S5. The top 1000 SNPs associated with acquiring cervical and vaginal HPV infections. Table S6. Positions of genes associated with the top 30 SNPs associated with acquiring HPV. Table S7. Genes associated with the top 500 SNPs associated with acquiring HPV, developing fully persistent and partially/fully persistent HPV infections, respectively. Table S8. Full list of biological pathways associated with acquiring HPV. Table S9. The top 1000 SNPs associated with developing persistent HPV infections. Table S10. Positions of genes associated with the top 30 SNPs associated with developing persistent HPV infections. Table S11. Full list of biological pathways associated with developing persistent HPV infections. Table S12. The top 1000 SNPs associated with developing partially/fully persistent HPV infections. Table S13. Positions of genes associated with the top 30 SNPs associated with partially/fully persistent HPV infections. Table S14. Full list of biological pathways associated with developing partially/fully persistent HPV infections.

Supplementary Material 5: Additional file 5. Supplementary methods.

Supplementary Material 6: Additional file 6. Supplementary gene and reference list.

## Data Availability

The full raw GWAS data will be submitted to the European Genome-Phenome Archive.

## References

[1] Jedy-Agba E, Joko WY, Liu B, Buziba NG, Borok M, Korir A, et al. Trends in cervical cancer incidence in sub-Saharan Africa. Br J Cancer. 2020;123(1):148–54. 10.1038/s41416-020-0831-9. PMC7341858 32336751

[2] De Vuyst H, Alemany L, Lacey C, Chibwesha CJ, Sahasrabuddhe V, Banura C, et al. The burden of human papillomavirus infections and related diseases in sub-saharan Africa. Vaccine. 2013;31(Suppl 5):F32–46. 10.1016/j.vaccine.2012.07.092. PMC4144870 24331746

[3] Uwamungu S, Andersson M, Zewdie BN, Muvunyi CM, Bienvenu E, Hasseus B, et al. High Concordance between Vaginal Samples and Cervical Samples of Human Papillomavirus in Women Living with HIV in Rwanda. BMC Infect Dis. 2025;25(1):527. 10.1186/s12879-025-10840-7. PMC12001398 40234747

[4] Andersson M, Zewdie BN, Mumena C, Thulin EA, Woldeamanuel GG, Hasseus B, et al. Prevalence of cervical human papillomavirus infections and cytological abnormalities in asymptomatic women living with HIV in Addis Ababa, Ethiopia. Sci Rep. 2026;16(1). 10.1038/s41598-026-64488-7. PMC13427740 42538348

[5] Sayinzoga F, Umulisa MC, Sibomana H, Tenet V, Baussano I, Clifford GM. Human papillomavirus vaccine coverage in Rwanda: A population-level analysis by birth cohort. Vaccine. 2020;38(24):4001–5. 10.1016/j.vaccine.2020.04.021. PMC7221340 32336599

[6] Chizyuka N, Crawford E, Hebert KS, Gaju S, Mumukunde I, Dusengimana JMV, et al. Lessons from the design and rollout of an electronic medical record system for cervical cancer screening in Rwanda. Glob Health Sci Pract. 2024;12(3). 10.9745/GHSP-D-23-00469. PMC11216700 38670797

[7] Mukanyangezi MF, Rugwizangoga B, Manzi O, Rulisa S, Hellstrand K, Tobin G, et al. Persistence rate of cervical human papillomavirus infections and abnormal cytology in Rwanda. HIV Med. 2019;20(7):485–95. 10.1111/hiv.12782. 31318136

[8] Stelzle D, Tanaka LF, Lee KK, Ibrahim Khalil A, Baussano I, Shah ASV, et al. Estimates of the global burden of cervical cancer associated with HIV. Lancet Glob Health. 2021;9(2):e161–9. 10.1016/S2214-109X(20)30459-9. PMC7815633 33212031

[9] Dusingize JC, Murenzi G, Muhoza B, Businge L, Remera E, Uwinkindi F, et al. Cancer risk among people living with Human Immuodeficiency Virus (HIV) in Rwanda from 2007 to 2018. Int J Cancer. 2024. 10.1002/ijc.35091. PMC12173455 39128948

[10] Brophy J, Bitnun A, Alimenti A, Lapointe N, Samson L, Read S, et al. Immunogenicity and Safety of the Quadrivalent Human Papillomavirus Vaccine in Girls Living With HIV. Pediatr Infect Dis J. 2018;37(6):595–7. 10.1097/INF.0000000000001874. 29278613

[11] Bienvenu E, Mukanyangezi MF, Rulisa S, Martner A, Hasseus B, Vorontsov E, et al. Changes in the proteome in the development of chronic human papillomavirus infection—a prospective study in HIV positive and HIV negative Rwandan women. Cancers (Basel). 2021;13(23). 10.3390/cancers13235983. PMC8656715 34885095

[12] Ramachandran D, Schurmann P, Mao Q, Wang Y, Bretschneider LM, Speith LM, et al. Association of genomic variants at the human leukocyte antigen locus with cervical cancer risk, HPV status and gene expression levels. Int J Cancer. 2020;147(9):2458–68. 10.1002/ijc.33171. 32580243

[13] Adebamowo SN, Adeyemo AA, Rotimi CN, Olaniyan O, Offiong R, Adebamowo CA, et al. Genome-wide association study of prevalent and persistent cervical high-risk human papillomavirus (HPV) infection. BMC Med Genet. 2020;21(1):231. 10.1186/s12881-020-01156-1. PMC7682060 33225922

[14] Adebamowo SN, Adeyemo A, Adebayo A, Achara P, Alabi B, Bakare RA, et al. Genome, HLA and polygenic risk score analyses for prevalent and persistent cervical human papillomavirus (HPV) infections. Eur J Hum Genet. 2024;32(6):708–16. 10.1038/s41431-023-01521-7. PMC11153215 38200081

[15] Uwamungu S, Nigussie B, Muvunyi CM, Hasseus B, Andersson M, Giglio D. Prevalence of human papillomavirus in different mucous membranes in HIV concordant couples in Rwanda. Viruses. 2023;15(4). 10.3390/v15041005. PMC10145982 37112984

[16] Gunderson KL, Steemers FJ, Lee G, Mendoza LG, Chee MS. A genome-wide scalable SNP genotyping assay using microarray technology. Nat Genet. 2005;37(5):549–54. 10.1038/ng1547. 15838508

[17] Steemers FJ, Chang W, Lee G, Barker DL, Shen R, Gunderson KL. Whole-genome genotyping with the single-base extension assay. Nat Methods. 2006;3(1):31–3. 10.1038/nmeth842. 16369550

[18] Purcell S, Neale B, Todd-Brown K, Thomas L, Ferreira MA, Bender D, et al. PLINK: a tool set for whole-genome association and population-based linkage analyses. Am J Hum Genet. 2007;81(3):559–75. 10.1086/519795. PMC1950838 17701901

[19] Wu MC, Lee S, Cai T, Li Y, Boehnke M, Lin X. Rare-variant association testing for sequencing data with the sequence kernel association test. Am J Hum Genet. 2011;89(1):82–93. 10.1016/j.ajhg.2011.05.029. PMC3135811 21737059

[20] Ostensson M, Monten C, Bacelis J, Gudjonsdottir AH, Adamovic S, Ek J, et al. A possible mechanism behind autoimmune disorders discovered by genome-wide linkage and association analysis in celiac disease. PLoS ONE. 2013;8(8):e70174. 10.1371/journal.pone.0070174. PMC3732286 23936387

[21] Qin Y, Liang R, Lu P, Lai L, Zhu X. Depicting the Implication of miR-378a in Cancers. Technol Cancer Res Treat. 2022;21:15330338221134385. 10.1177/15330338221134385. PMC9608056 36285472

[22] Honegger A, Schilling D, Bastian S, Sponagel J, Kuryshev V, Sultmann H, et al. Dependence of intracellular and exosomal microRNAs on viral E6/E7 oncogene expression in HPV-positive tumor cells. PLoS Pathog. 2015;11(3):e1004712. 10.1371/journal.ppat.1004712. PMC4356518 25760330

[23] Zou C, Lyu Y, Jiang J, Cao Y, Wang M, Sang C, et al. Use of peripheral blood transcriptomic biomarkers to distinguish high-grade cervical squamous intraepithelial lesions from low-grade lesions. Oncol Lett. 2020;20(3):2280–90. 10.3892/ol.2020.11779. PMC7403635 32765790

[24] Kraus I, Driesch C, Vinokurova S, Hovig E, Schneider A, von Knebel Doeberitz M, et al. The majority of viral-cellular fusion transcripts in cervical carcinomas cotranscribe cellular sequences of known or predicted genes. Cancer Res. 2008;68(7):2514–22. 10.1158/0008-5472.CAN-07-2776. 18381461

[25] Liu L, Ren M, Han S, Sun L, Zhu L. Expression level and clinical significance of low-density lipoprotein receptor-related protein 1B gene in cervical squamous cell carcinoma. Int J Clin Exp Pathol. 2018;11(3):1701–6. PMC6958098 31938272

[26] GTEX release 6. https://www.gtexportal.org/home/ Accessed.

[27] Middha P, Wang X, Behrens S, Bolla MK, Wang Q, Dennis J, et al. A genome-wide gene-environment interaction study of breast cancer risk for women of European ancestry. Breast Cancer Res. 2023;25(1):93. 10.1186/s13058-023-01691-8. PMC10411002 37559094

[28] Sorensen JK, Jensen A, Zimakoff AC, Vittrup DM, Malon M, Kim S, et al. Genetic associations with measles PRNT and IgG antibody response to MMR vaccination in 6- and 15-month-old children. Vaccine. 2025;50:126788. 10.1016/j.vaccine.2025.126788. 39914254

[29] Obermayr E, Sanchez-Cabo F, Tea MK, Singer CF, Krainer M, Fischer MB, et al. Assessment of a six gene panel for the molecular detection of circulating tumor cells in the blood of female cancer patients. BMC Cancer. 2010;10:666. 10.1186/1471-2407-10-666. PMC3013085 21129172

[30] Kawashita S, Matsuda K, Kondo H, Kitajima Y, Hasegawa Y, Shimada T, et al. Significance of p53-Binding Protein 1 Nuclear Foci in Cervical Squamous Intraepithelial Lesions: Association With High-Risk Human Papillomavirus Infection and P16(INK4a) Expression. Cancer Control. 2020;27(1):1073274819901170. 10.1177/1073274819901170. PMC7044491 32077330

[31] Loonen AJM, Verhagen F, Luijten-de Vrije I, Lentjes-Beer M, Huijsmans CJ, van den Brule AJC. Vaginal dysbiosis seems associated with hrHPV infection in women attending the Dutch Cervical Cancer Screening Program. Front Cell Infect Microbiol. 2024;14:1330844. 10.3389/fcimb.2024.1330844. PMC10965536 38544527

[32] Hu Z, Zhu D, Wang W, Li W, Jia W, Zeng X, et al. Genome-wide profiling of HPV integration in cervical cancer identifies clustered genomic hot spots and a potential microhomology-mediated integration mechanism. Nat Genet. 2015;47(2):158–63. 10.1038/ng.3178. 25581428

[33] Liu CX, Musco S, Lisitsina NM, Yaklichkin SY, Lisitsyn NA. Genomic organization of a new candidate tumor suppressor gene, LRP1B. Genomics. 2000;69(2):271–4. 10.1006/geno.2000.6331. 11031110

[34] Liu Z, Teng L, Gao L, Wang H, Su Y, Li J. The role of eukaryotic translation initiation factor 5A-1 (eIF5A-1) gene in HPV 16 E6 induces cell growth in human cervical squamous carcinoma cells. Biochem Biophys Res Commun. 2018;504(1):6–12. 10.1016/j.bbrc.2018.08.018. 30170728

[35] Gordon DE, Jang GM, Bouhaddou M, Xu J, Obernier K, White KM, et al. A SARS-CoV-2 protein interaction map reveals targets for drug repurposing. Nature. 2020;583(7816):459–68. 10.1038/s41586-020-2286-9. PMC7431030 32353859

[36] Murenzi G, Tuyisenge P, Kanyabwisha F, Munyaneza A, Muhoza B, Kubwimana G, et al. Type-specific persistence, clearance and incidence of high-risk HPV among screen-positive Rwandan women living with HIV. Infect Agent Cancer. 2021;16(1):16. 10.1186/s13027-021-00355-6. PMC7893720 33608036

[37] Dyer SC, Austine-Orimoloye O, Azov AG, Barba M, Barnes I, Barrera-Enriquez VP, et al. Ensembl 2025. Nucleic Acids Res. 2025;53(D1):D948–57. 10.1093/nar/gkae1071. PMC11701638 39656687

