# Supplementary material for: Genome-wide association study of human papillomavirus infection and persistence in women living with HIV in Rwanda
Source: Infect Agent Cancer. 2026 Oct 26;21:72. doi: 10.1186/s13027-026-00815-x (PMC13700139; doi:10.1186/s13027-026-00815-x)
Supplement: Supplementary file 2 [file 13027_2026_815_MOESM2_ESM.pdf]

University of Rwanda

University of Gothenburg
Gothenburg
Sweden

Cohort A. Prevalence of HPV co-infections in HIV- and HIV+ Rwandan women

This questionnaire is part of a research project to investigate how common HPV infection is in the uterine cervix among Ethiopian women. Moreover, since HPV may occur in other mucous membranes such as of the anus and the mouth, we want to assess whether HPV infections exist in these areas too. The project is initiated from the Department of Oncology, the Sahlgrenska Academy at the University of Gothenburg, Gothenburg, Sweden. Principal investigator is Associate Professor Daniel Giglio (daniel.giglio@pharm.gu.se). The participation in this study is voluntary. Participation is anonymous and participants will be given a unique code linked to their individual questionnaire. Coding linking the participant to their individual questionnaire will only be known to the research group and will not be given to anyone outside of the research group./ Ibi bibazo ni ibijyanye n'ubushakashatsi buzareba ubwiganze bw'agakoko ka HPV gatera uburwayi inkondo yykondo mu bagore babanyarwandakazi, nubwo aka gakoko gashobora kuba mu bindi bice by'yice by byorohereye nko mu nnyo, mu kanwa, turifuza kuzareba niba aka gakoko ka HPV katatera uburwayi no muri ibyo bice bindi. Ubu bushakashatsi bwatangijwe nnatangi rireba ibya cancer muri Sweden, University of Gothenburg, uhagarariye ubushakashatsi aka ari Umwarimu muri iyo Kaminuza Associate Professor Daniel Giglio (daniel.giglio@pharm.gu.se). Kwitabira ubu bushakashatsi ni ubushake. Abazitabira ubushakashatsi amazina yabo ntazagaragazwa, bazahabwa umubare wwubare ubahuza n'nahuza nn babarijweho. Umubare wwubare uhuza uwemeye kuba mu bushashatsi nnshashatsi yabarijweho, uzamenywa gusa nnsa nnwa ryya nnwa gusa n gusa, nta wundi muntu wo hanze yabo uzawumenya.

Questionnaire identification number/Umubare uranga ifishi y'ibibazo mu bushakashatsi:

Participant Identification number in the study/Umubare uranga uwagize uruhare mu gusubiza:

Interviewer Name/ Amazina y'ubaza ....

Interview Date/Itariki y'ibazwa...

I will ask some questions that will be recorded in this notebook. I must say that everything you answer will be strictly confidential, and the information gathered from you will be used only in scientific reports, without personal identification. / *Ngiye kukubaza ibibazo maze ibisubizo byandikwe muri aka gatabo.Ndakwizeza neza ko abari abari muri ubu bushakashatsi bose bazagira ibanga ku bisubizo uri butange kandi ko bizakoreshwa gusa mu bijyanye n'intego z'ubu bushakashatsi.*

1. BACKGROUND INFORMATION/ Amakuru y'ibanze

1A. What is your age? Ufite imyaka ingahe y'amavuko?…………

1B.Gender/Igitsinacyawe

A.Male/Gabo

B.Female/Gore

1C. What is your highest educational level attained? *Ni uruhe rwego rwanyuma rw'amashuri warangije? (Hitamo igisubizo kimwe)*

A. None/ Ntiyize
B. Incomplete primary school/ Ntiyarangije abanza
C. Complete primary school/ Yarangije abanza
D. Secondary school incomplete/ Ntiyarangije ayisumbuye
E. Complete Secondary school/ Yarangije ayisumbuye
F. Incomplete University/Ntiyarangije kaminuza
G. Complete University/ Yarangije kaminuza
H. Others (Specify) / Ibindi (Sobanura)

1D. Occupation/ Umurimo ukora

A. Farming/ Umuhinzi mworozi
B. Civil servant/ Umukozi wa leta
C. Business/Umucuruzi
D. Community health worker/ Akora mu buzima
E. Unemployed/Ntabwo akora
E. Others (specify)/ Ibindi (Sobanura)...

1E. Marital status/ Irangamimerere

A. Is married/Arubatse
B. Has a male partner but not married/ Afite umugabo batasezeranye
C. Has a female partner but not married/ Afite umugore batasezeranye
D. Is separated/divorced/Yatandukanye nuwo bashakanye
E. Is widow/Ni umupfakazi
F. Is single (never been married or lived with a partner)/ Ni ingaragu ntiyigeze ashaka cg ngo abane n'umugabo cg umugore

2. MEDICAL HISTORY/Amateka y'ubuvuzi

2A. Have you or had any serious diseases in the past two year? / Waba warigeze ugira indwara ikomeye mu myaka ibiri ishize?

A. Yes/Yego
B. No/Oya

If, yes, please specify/Niba ari yego ni iyihe

Cardiovascular disease/uburwayi bw'umutima?
Cancer/Kanseri?
Rheumatic disease/Indwara zo mu ngingo?
Other serious disease/ Indi ndwara ikomeye

2B. Do you take any medication regularly? /Hari imiti ufata ku buryo buhoraho?

A. Yes/ Yego
B. No/Oya

Conventional drugs/Imiti ya kizungu: ……………

Traditional drugs/Imiti ya gakondo: …………….

2C. Do you have any allergy/hypersensitivity? /Hari imiti umubiri wawe ugiraho ikibazo?

A. Yes/ Yego
B. No/ Oya

If yes, please specify/Niba ari yego ni iyihe? **………………………..**

3. HABITS/ IMICO

3A. Are you a smoker? /Unywa itabi?

A. No, I have never been a smoker/Oya, Sindarinywa na rimwe
B. Yes, I am currently a smoker/ Yego, Ubu ndarinywa
C. I am an ex-smoker/ Narariretse

3B. How often do you drink alcohol? /Unywa inzoga inshuro zingahe?
A. Never/seldom/ Nta na rimwe
B. Once a month/ Rimwe mu kwezi
C. Once a week/ Rimwe mu Cyumweru
D. Several times a week/ Inshuro nyinshi mu Cyumweru

4. SEXUAL AND REPRODUCTIVE LIVES/ UBUZIMA BW'IMYORORОКЕRE

We are currently examining virus that can be transmitted through sexual contact. Therefore, we would like to ask you some questions about your sexual and reproductive life. /Turimo gupima agakoko ka virusi gashobora gukwirakwizwa binyuze mu mibonano mpuzabitsina. Turifuza kubabaza ibibazo ku bijyanye n'ubuzima bw'imyororokere yanyu.

4B. Have you had additional new sexual partners in the past two years? / Ese hari abantu bashya biyongereye kubo mwari mwarakoranye imibonano mpuzabitsina, mu myaka ibiri ishize?

A. Yes/ yego
B. No/oya

If yes, specify the number? Niba ari yego vuga umubare…………………………….........

4C. Have you had new live birth in this past two years? / Wageze ubyara umwana uriho mu myaka ibiri ishize?

Specify number of live births in this past two years ________________________

4D. How many spontaneous and/or voluntary abortions have you had in the past two years? / Waba warakuyemo inda zingahe ku bushake cg bigutunguye muri iyi myaka ibiri ishize?

Specify the number of abortions in the past two years ________________________

4E. Have you had syphilis these past two years? /Waba wararwaye mburugu mu myaka ibiri ishize?

a. No/ Oya
b. Yes/ Yego
c. I don´t know/ Ntabwo mbizi

4F. Have you had gonorrhea these past two years? /Waba wararwaye imitezi mu myaka ibiri ishize?

a. No/ Oya
b. Yes/ Yego
c. I don´t know/ Ntabwo mbizi

4G. Have you had chlamydia these past two years? / Waba wararwaye kalamidiya mu myaka ibiri ishize?

a. No/ Oya
b. Yes/ Yego
c. I don´t know/ Ntabwo mbizi

4H. Have you had other sexually transmitted diseases in the past two years? / Waba wararwaye izindi ndwara zandurira mu mibonano mpuzabitsina mu myaka ibiri ishize?

Specify/Zivuge...................................

4J. Are you using a contraceptive method now? Waba ukoresha uburyo bwo kuboneza urubyaro?

Yes/ Yego

No/ Oya

If yes, go to question 4K/ Niba ari yego jya ku kibazo cya 4K

4K. Can you specify the method (multiple selections can be made)? / Watubwira uburyo ukoresha bwo kuboneza urubyaro ubu (niba ukoresha uburenze bumwe buvuge bwose)?

a. female sterilization/ Kwifungisha burundu ku mugore
b. male sterilization/ Gufunga burundu ku mugabo
c. Contraceptive implant/ Agapira
d. Contraceptive injectable/ Urushinge
e. Contraceptive pill/ Ibinini
f. Male condom/ Gukoresha agakingirizo
g. Rhytm method monitoring fertility/ Kubara
h. Withdrawal of penis before ejaculation/ Kwiyakana
i. Other methods. Specify/ Ubundi buryo_________________________
j. I am not using a contraceptive method. / Nta buryo na bumwe nkoresha

Thank you for your time and effort! / Murakoze ku gihe n'ubwitange bwanyu!

To be completed by a physician from patient file / Biruzuzwa n'umuganga

Patient's HIV Clinical staging__________________________

19. CD4 + Count (cells/dl)

A. < 200
B. 200-500
C. > 500

20. HIV viral load (copies/ml)

A. < 400
B. 400-5000
C. >5000

Thank you for your time and effort!
