# Supplementary material for: Genome-wide association study of human papillomavirus infection and persistence in women living with HIV in Rwanda
Source: Infect Agent Cancer. 2026 Oct 26;21:72. doi: 10.1186/s13027-026-00815-x (PMC13700139; doi:10.1186/s13027-026-00815-x)
Supplement: Supplementary file 3 [file 13027_2026_815_MOESM3_ESM.docx]

**Supplementary figures**

Figure S1. Study flow chart.

Figure S2. Study design.

Figure S3. Q-Q plot for the cleared versus fully persistent HPV CMH GWAS.

Observed versus expected -log10(P) values for the cleared-versus-fully-persistent cluster-stratified CMH GWAS. The genomic inflation factor was λGC = 1.010. Most of the distribution closely followed the null expectation, with upward deviation in the upper tail. Abbreviations: CMH, Cochran-Mantel-Haenszel; GWAS, genome-wide association study; HPV, human papillomavirus; Q-Q, quantile-quantile; λGC, genomic inflation factor.

Figure S4. Q-Q plot for the cleared versus partially/fully persistent HPV CMH GWAS.

Observed versus expected -log10(P) values for the cleared-versus-partially/fully persistent cluster-stratified CMH GWAS. The genomic inflation factor was λGC = 1.064. The distribution showed close agreement with the null expectation across most P values, with modest deviation in the extreme upper tail. Abbreviations: CMH, Cochran-Mantel-Haenszel; GWAS, genome-wide association study; HPV, human papillomavirus; Q-Q, quantile-quantile; λGC, genomic inflation factor.
