# Supplementary material for: Genome-wide association study of human papillomavirus infection and persistence in women living with HIV in Rwanda
Source: Infect Agent Cancer. 2026 Oct 26;21:72. doi: 10.1186/s13027-026-00815-x (PMC13700139; doi:10.1186/s13027-026-00815-x)
Supplement: Supplementary file 4 [file 13027_2026_815_MOESM5_ESM.docx]

*Real-time quantitative PCR*

Nucleic acids (DNA) were extracted from 200 µl of each specimen using the DNA Isolation I kit and the MagNA Pure LC instrument (Roche Molecular, Mannheim, Germany). The extracted nucleic acids were eluted to a volume of 100 µl, and 5 µl was then used for each qPCR reaction. qPCR was conducted in eight parallel reactions of 20 µl each, containing oligonucleotides listed in Table S1, and Universal Mastermix (Applied Biosystems, Foster City, CA, USA). The assay ran for 45 cycles (15 seconds at 95°C, 60 seconds at 58°C) following an initial 10-minute denaturation at 95°C, using a QuantStudio 6 384-well system (Applied Biosystems). To confirm the performance of each multiplex reagent mixture, pUC57 plasmids with targeted HPV sequence inserts, synthesized by GenScript Corp. (Piscataway, NJ, USA), were used. Positive results were defined by Ct values under 37 for samples. Participants were grouped according to whether they were negative for HPV at baseline and at follow-up, positive for HPV at baseline and had fully cleared all HPV types at follow-up, positive at baseline and cleared some of the HPV types at follow-up, positive at baseline and not cleared any HPV types at follow-up, and negative at baseline and contracted one or more HPV types at follow-up.

For the analysis of biomarker gene expressions, complementary DNA (cDNA) was prepared directly from cervical samples, using bovine serum albumin (BSA) as evaluated by Svec et al. [1], and the High-Capacity cDNA Reverse Transcription Kit (Thermo Fisher Scientific), excluding the RNase inhibitor. To prepare 20 µl of cDNA samples, we used 13.2 µl of unextracted sample in Ripa buffer, 1.5 µl of BSA, and 5.8 µl of High-Capacity cDNA Reverse Transcription Kit. After obtaining the cDNA samples, a qPCR on target gene assays and five housekeeping genes (details on primers are displayed in Table S1) mixed with universal master mix without UNG was performed using the QuantStudio 12k flex, 384-well system (Applied Biosystems). CT values defining expression and participant groups are as described above.

*Cytology*

Cytological samples were classified in accordance with the 2014 Bethesda System as negative for squamous intraepithelial lesion or malignancy (NILM), atypical squamous cells of undetermined significance (ASCUS), low-grade squamous intraepithelial lesion (LSIL), atypical squamous cells cannot exclude high-grade squamous intraepithelial lesion (ASC-H), atypical glandular cells (AGC), endocervical adenocarcinoma in situ (AIS), high-grade squamous intraepithelial lesion (HSIL), and squamous cell carcinoma (SCC). Pap smears were assessed by a well-experienced pathologist/cytologist (BNZ).

*GWAS*

We used PLINK (v1.9 and v2.0) [2] to perform association analysis including Mantel–Haenszel tests for stratified case–control data, and to group individuals into more homogeneous clusters to account for potential population substructure within the study cohort (see more details on this analysis below). Three different GWAS case-control comparisons were made; i) ever versus never infected with HPV, ii) persistent HPV versus total clearance, and iii) partially persistent and fully persistent HPV versus total clearance. Genes surrounding the top 30 associated SNPs in each analysis (+/- 200 kb), were used to search in the PubMed database for connections to HPV (search term “HPV” AND “gene name”; “cervical cancer” AND “gene name”). All QC steps were executed under a Linux/WSL environment. Initial variant- and sample‑level filtering removed individuals with missingness > 5% and SNPs with call rate <95%. Hardy Weinberg Equilibrium (HWE) was evaluated and calculated for each variant. Sex discrepancies were evaluated using PLINK’s sex‑check module, and heterozygosity statistics were computed. Genomic inflation (λGC) was computed from the median χ² statistic, and Q–Q plots were generated to assess population stratification and model fit. Principal component analysis (PCA) was performed on LD‑pruned SNPs using PLINK2. Individuals deviating more than four standard deviations from the means of PC1 or PC2 were classified as population outliers and removed. The resulting dataset served as the genotype backbone for all downstream analyses.

We performed a cluster analysis that pairs individuals based on genetic similarity. This process involves using identity by state (IBS) clustering and genetic association analysis using the Cochran–Mantel–Haenszel (CMH) test as implemented in PLINK v1.9[2-5]. First, study participants were grouped into clusters representing genetically similar subpopulations. For each variant, the CMH test then evaluates the association between genotype and binary phenotype (case/control status) by constructing two-by-two contingency tables stratified by cluster. The test computes a weighted summary odds ratio over all strata, effectively adjusting for population stratification captured by the clustering. Only clusters containing both cases and controls were included in the analysis, as at least one individual from each outcome category is required per stratum to perform the test. This approach mitigates bias and inflation of association statistics caused by population structure or relatedness by conditioning on strata membership[6]. The CMH test provides a robust framework for detecting genuine genotype-phenotype associations within diverse or structured samples, improving reliability compared to unstratified analyses.

Rare‑variant association testing was performed using the Sequence Kernel Association Test (SKAT) framework [7]. Variants were restricted to minor allele frequency (MAF) < 0.05, ensuring that only rare and low‑frequency variants contributed to the gene‑based tests. For each gene, both SKAT and SKAT‑O p‑values were computed. SKAT evaluates the joint effect of multiple rare variants within the same gene, while allowing heterogeneous effect directions, whereas SKAT‑O adaptively combines SKAT with burden testing to maximize power across a range of genetic architectures. The top significant genes in SKAT/SKAT‑O were overlaid onto the GWAS Manhattan plot to highlight loci supported by this clustered rare‑variant signals.

To perform functional annotation, the most significant variants were exported and annotated using the Ensembl Variant Effect Predictor (VEP) with GRCh37. All VEP consequences were parsed, and the most functionally relevant annotation per variant was selected using a ranking scheme combining:

- VEP IMPACT (HIGH > MODERATE > LOW > MODIFIER)

- consequence class (stop gained > splice > frameshift > missense > regulatory > other)

Manhattan plots were generated using ggplot2 and ggrastr for efficient rendering of ~700k points. Genome‑wide/suggestive thresholds were indicated with dashed lines. Functional consequences (from VEP) were overlaid using shape encodings, and the 25 most significant genes and genes with functional SNPs were labeled using ggrepel.

**References**

1. Svec D, Andersson D, Pekny M, Sjoback R, Kubista M, Stahlberg A. Direct cell lysis for single-cell gene expression profiling. Front Oncol. 2013;3:274. doi: 10.3389/fonc.2013.00274.

2. Purcell S, Neale B, Todd-Brown K, Thomas L, Ferreira MA, Bender D, et al. PLINK: a tool set for whole-genome association and population-based linkage analyses. Am J Hum Genet. 2007;81(3):559-75. doi: 10.1086/519795.

3. W.G. C. Some methods for strengthening the common χ2 test. Biometrics. 1954;10:417–51. doi: 10.2307/3001616.

4. Purcell S: PLINK: Whole genome data analysis toolset. <http://pngu.mgh.harvard.edu/purcell/plink/> (2025). Accessed.

5. Mantel N, Haenszel W. Statistical aspects of the analysis of data from retrospective studies of disease. J Natl Cancer Inst. 1959;22(4):719-48.

6. Greenbaum G, Templeton AR, Bar-David S. Inference and Analysis of Population Structure Using Genetic Data and Network Theory. Genetics. 2016;202(4):1299-312. doi: 10.1534/genetics.115.182626.

7. Wu MC, Lee S, Cai T, Li Y, Boehnke M, Lin X. Rare-variant association testing for sequencing data with the sequence kernel association test. Am J Hum Genet. 2011;89(1):82-93. doi: 10.1016/j.ajhg.2011.05.029.
