# Supplementary material for: Genome-wide association study of human papillomavirus infection and persistence in women living with HIV in Rwanda
Source: Infect Agent Cancer. 2026 Oct 26;21:72. doi: 10.1186/s13027-026-00815-x (PMC13700139; doi:10.1186/s13027-026-00815-x)
Supplement: Supplementary file 5 [file 13027_2026_815_MOESM6_ESM.docx]

**Genes surrounding 19 of the 30 top SNPs associated with contraction of HPV infections previously reported with connection to HPV and HPV-related cancer**

*EMP2* (rs3087620 in exon 5)

*P53BP1* and *PDIA3* (rs8042868 in *CATSPER2* exon 3)

*EPHA5* (rs4146476)

*ELK3* and *LTA4H* (rs11837546 in *ELK3* intron 1)

*MCPH1* (rs7827867)

*BCAR3* (rs1011903 in intron 1)

*APRG1*, *ITGA9* and *GOLGA4* (rs169111 in *ITGA9* intron 3)

*BMPR1B* (rs6857589 in *UNC5* intron 3)

*RNF207*, *ICMT*, *HES2* and *TNFRSF25* (rs72854394/rs72858315 both in *ACOT7* intron 5)

*PTPRG* (rs9871209 in *CADPS* intron 27)

*COL5A1* and *RXRA* (rs7020291 in *LOC100506532* intron 7)

*FGF13-AS1*, *FGF13* and *MIR504* (rs5976172)

*RBFOX1* (rs12934429 in *RBFOX1* intron 1)

*GOT1*, *HPSE2* and *SLC25A28* (rs2494657 in *GOT1-DT* intron 2)

*MIR708* (rs662533 in *TENM4* intron 2)

*PTPRN2* (rs4716515 in intron 11)

*KALRN* (rs1444768 in intron 1)

*APCDD1L* (rs6015313 in *APCDD1L-DT* intron 6)

**Genes surrounding 14 of the 30 top SNPs associated with partially/fully persistent HPV infections previously reported with connection to HPV and HPV-related cancer**

*PPARGC1B* intron 1 and the *MIR378A* gene (rs13158830 and rs10463291)

*OXNAD1* near rs138078234, the missense variant in *RFTN1* exon 10)

*SLC31A1* and *SLC46A2* (rs10981622 in *ZFP37* intron 1)

*GRPEL1* (rs116205824 in *GRPEL1*, the 3 prime UTR variant in exon 4)

*DOCK9* (rs8000767 in *SLC15A1* intron 17)

*PINK1* and *DDOST* (rs75357225 in *EIF4G3* intron 30)

LDL Receptor Related Protein 1B (*LRP1B*) (rs79417794 in *LRP1B* intron 66)

*DNMT3L* and *ICOSLG* (rs34219046, the missense variant in *AIRE* exon 5)

*DNMT1*, *PIN1*, and *COL5A3* (rs3786701 in *RDH8* intron 1)

*RBFOX1* (rs79585542 in intron 11)

*TEK* and *PLAA* (rs11789885 in *TEK* intron 1)

*RSBN1* (rs144740096 in *OLFML3* exon 3)

*TXNDC11* and *BCAR4* (rs117131967)

**Genes surrounding 23 of the 30 top SNPs associated with partially/fully persistent HPV infections previously reported with connection to HPV and HPV-related cancer**

*LINC00861* (rs7840807 in *LINC00861* intron 3)

*MIR888* and *MIR892B* (rs56322118 in *SLITRK2* intron 1/rs3810704 in *SLITRK2* exon 5)

*BID* (rs5992749 in *ATP6V1E1* intron 8); *MECOM* (rs13324022 in *MECOM* intron 2)

*DNMT1*, *TYK2*, *ICAM1*, *S1PR2*, *ICAM3* and *ICAM5* (rs12984733)

*DLG2* (rs7120512 in *DLG2* intron 6)

*LSP1*, *H19* and *CTSD* (rs116033749)

*OXNAD1* (rs62236345 in *RFTN1* intron 3)

*SLC8A1* (rs17026003 in *SLC8A1* intron 1)

*NFIA* (rs535381 in *LOC101926964* intron 1)

*ACVRL1*, *SCN8A*, *NR4A1* and *ACVR1B* (rs61913988)

*SOX30* and *CYFIP2* (rs72811321 in *ADAM19* intron 5)

*GOLM1* (rs4620358)

*PRKG1* (rs2339681 in *PRKG1* intron 1)

*ATP10A* (rs74644757 in *ATP10A* intron 16)

*TFAP2B* (rs4715215)

*CCND3* (rs9471809 in *MRPS10* exon 1)

*DMD* (rs7065982 intron 44)

*BAIAP3*, *UBE2I* and *SSTR5-AS1* (rs75538880 in *CACNA1H* intron 27)

*CRYAA*, *U2AF1*, *LINC00313*, *LINC00319* and *SIK1* (rs9283037)

*RET* (rs7068646)

*PTCH1* (rs817184)

**Genes close to SNPs in alphabetic order**

*ACVR1B* (1); *ACVRL1* (2); *APCDD1L* (3); *APRG1* (4);*ATP10A* (5)

*BAIAP3* (6); *BCAR3* (7); *BCAR4* (8); *BID* (9); *BMPR1B* (10)

*CCND3* (11); *COL5A1* (12); *COL5A3* (13); *CRYAA* (14); *CTSD* (15); *CYFIP2* (16)

*DDOST* (17); *DLG2* (18); *DMD* (19); *DNMT1* (20); *DNMT3L* (21); *DOCK9* (22)

*EMP2* (23); *ELK3* (24); *EPHA5* (25)

*FGF13* (26); *FGF13-AS1* (27)

*GOLGA4* (28); *GOLM1* (29); *GOT1* (30); *GRPEL1* (31)

*H19* (32); *HES2* (33);*HPSE2* (34)

*ICAM1* (35); *ICAM3* (36); *ICAM5* (37); *ICMT* (38); *ICOSLG* (39); *ITGA9* (4)

*KALRN* (40)

*LINC00313* (41); *LINC00319* (42); *LINC00861* (43); *LRP1B* (44); *LSP1* (45); *LTA4H* (46)

*MCPH1* (47); *MECOM* (48); *MIR378A* (49); *MIR504* (50); *MIR708* (51); *MIR888* (52); *MIR892B* (52)

*NFIA* (53); *NR4A1* (54)

*OXNAD1* (45)

*PDIA3* (55); *PIN1* (56); *PINK1* (57); *PLAA* (58); *PRKG1* (59); *PTCH1* (60); *PTPRG* (61); *PTPRN2* (62)

*RBFOX1* (63); *RET* (64); *RSBN1* (65); *RNF207* (66); *RXRA* (67)

*SCN8A* (68); *SIK1* (69); *S1PR2* (70); *SLC8A1* (71); *SLC25A28* (72); *SLC31A1* (73); *SLC46A2* (74); *SOX30* (75); *SSTR5-AS1* (76)

*TEK* (77); *TFAP2B* (78); *TNFRSF25* (79); *TP53BP1* (80); *TXNDC11* (81); *TYK2* (82)

*U2AF1* (83); *UBE2I* (84)

**References**

1. Yuan M, Cheng J, Liu Y, et al. [screening and functional analysis of microrna expression in hpv16-positive squamous carcinoma of the cervix  in the uygur of southern xinjiang]. *Zhong Nan Da Xue Xue Bao Yi Xue Ban* 2015;40:701-9.

2. Psyrri A, Seiwert TY, Jimeno A. Molecular pathways in head and neck cancer: Egfr, pi3k, and more. *Am Soc Clin Oncol Educ Book* 2013:246-55.

3. Chen A, Jing W, Qiu J, et al. Prediction of cervical cancer outcome by identifying and validating a nad+ metabolism-derived gene signature. *J Pers Med* 2022;12.

4. Senchenko VN, Kisseljova NP, Ivanova TA, et al. Novel tumor suppressor candidates on chromosome 3 revealed by noti-microarrays in cervical cancer. *Epigenetics* 2013;8:409-20.

5. El-Zein M, Cheishvili D, Gotlieb W, et al. Genome-wide DNA methylation profiling identifies two novel genes in cervical neoplasia. *Int J Cancer* 2020;147:1264-1274.

6. Li W, Lei W, Chao X, et al. Genomic alterations caused by hpv integration in a cohort of chinese endocervical adenocarcinomas. *Cancer Gene Ther* 2021;28:1353-1364.

7. Jeannot E, Harle A, Holmes A, et al. Nuclear factor i x is a recurrent target for hpv16 insertions in anal carcinomas. *Genes Chromosomes Cancer* 2018;57:638-644.

8. Cancer Genome Atlas Research N, Albert Einstein College of M, Analytical Biological S, et al. Integrated genomic and molecular characterization of cervical cancer. *Nature* 2017;543:378-384.

9. Heber N, Kuhn BJ, Strobel TD, et al. The impact of cycling hypoxia on the phenotype of hpv-positive cervical cancer cells. *J Med Virol* 2023;95:e29280.

10. Prates J, Franco-Salla GB, Dinarte Dos Santos AR, et al. Anxa1ac(2)(-)(2)(6) peptide reduces id1 expression in cervical carcinoma cultures. *Gene* 2015;570:248-54.

11. Raina R, Afroze N, Kedhari Sundaram M, et al. Chrysin inhibits propagation of hela cells by attenuating cell survival and inducing apoptotic pathways. *Eur Rev Med Pharmacol Sci* 2021;25:2206-2220.

12. Duan X, Ye D, Yuan J, et al. Col5a1 overexpression correlates with poor prognosis in human cervical cancer. *Int J Biol Markers* 2024;39:265-273.

13. Ganguly P, Ganguly N. Transcriptomic analyses of genes differentially expressed by high-risk and low-risk human papilloma virus e6 oncoproteins. *Virusdisease* 2015;26:105-16.

14. Naru J, Aggarwal R, Mohanty AK, et al. Identification of differentially expressed proteins in retinoblastoma tumors using mass spectrometry-based comparative proteomic approach. *J Proteomics* 2017;159:77-91.

15. Wozniak B, Mila-Kierzenkowska C, Wozniak A, et al. The effect of combined therapy on activity of cathepsin d and alpha-1-antitrypsin in the blood serum of women with cervical cancer. *Eur J Gynaecol Oncol* 2008;29:617-9.

16. Li Y, Song X, Liu L, et al. Nuak2 silencing inhibits the proliferation, migration and epithelial‑to‑mesenchymal transition of cervical cancer cells via upregulating cyfip2. *Mol Med Rep* 2021;24.

17. Mei J, Pan L, Huang M, et al. Ddost is associated with tumor immunosuppressive microenvironment in cervical cancer. *Discov Oncol* 2024;15:69.

18. Hu Z, Zhu D, Wang W, et al. Genome-wide profiling of hpv integration in cervical cancer identifies clustered genomic hot spots and a potential microhomology-mediated integration mechanism. *Nat Genet* 2015;47:158-63.

19. Femi OF. Genetic alterations and pik3ca gene mutations and amplifications analysis in cervical cancer by racial groups in the united states. *Int J Health Sci (Qassim)* 2018;12:28-32.

20. Dutta P, Basu M, Roy A, et al. High nuclear expression of dnmt1 in correlation with inactivation of tet1 portray worst prognosis among the cervical carcinoma patients: Clinical implications. *J Mol Histol* 2023;54:89-102.

21. Gokul G, Ramakrishna G, Khosla S. Reprogramming of hela cells upon dnmt3l overexpression mimics carcinogenesis. *Epigenetics* 2009;4:322-9.

22. Ruiz-Lafuente N, Minguela A, Muro M, et al. The roles of cdc42 and rac1 in the formation of plasma membrane protrusions in cancer epithelial hela cells. *Mol Biol Rep* 2021;48:4285-4294.

23. Obermayr E, Sanchez-Cabo F, Tea MK, et al. Assessment of a six gene panel for the molecular detection of circulating tumor cells in the blood of female cancer patients. *BMC Cancer* 2010;10:666.

24. Dai G, Ou J, Wu B. A predictive study of metabolism reprogramming in cervical carcinoma. *Ann Transl Med* 2022;10:414.

25. Kori M, Yalcin Arga K. Potential biomarkers and therapeutic targets in cervical cancer: Insights from the meta-analysis of transcriptomics data within network biomedicine perspective. *PLoS One* 2018;13:e0200717.

26. Yu H, Wang H, Qie A, et al. Fgf13 enhances resistance to platinum drugs by regulating hctr1 and atp7a via a microtubule-stabilizing effect. *Cancer Sci* 2021;112:4655-4668.

27. Cui H, Ma R, Hu T, et al. Bioinformatics analysis highlights five differentially expressed genes as prognostic biomarkers of cervical cancer and novel option for anticancer treatment. *Front Cell Infect Microbiol* 2022;12:926348.

28. Senchenko V, Liu J, Braga E, et al. Deletion mapping using quantitative real-time pcr identifies two distinct 3p21.3 regions affected in most cervical carcinomas. *Oncogene* 2003;22:2984-92.

29. Ge X, Gu Y, Li D, et al. Knockdown of lncrna pcat1 enhances radiosensitivity of cervical cancer by regulating mir-128/golm1 axis. *Onco Targets Ther* 2020;13:10373-10385.

30. Cai L, Hu C, Yu S, et al. Identification and validation of a six-gene signature associated with glycolysis to predict the prognosis of patients with cervical cancer. *BMC Cancer* 2020;20:1133.

31. Zou C, Lyu Y, Jiang J, et al. Use of peripheral blood transcriptomic biomarkers to distinguish high-grade cervical squamous intraepithelial lesions from low-grade lesions. *Oncol Lett* 2020;20:2280-2290.

32. Zhong Q, Lu M, Yuan W, et al. Eight-lncrna signature of cervical cancer were identified by integrating DNA methylation, copy number variation and transcriptome data. *J Transl Med* 2021;19:58.

33. Katoh M, Katoh M. Identification and characterization of human hes2, hes3, and hes5 genes in silico. *Int J Oncol* 2004;25:529-34.

34. Marques RM, Focchi GR, Theodoro TR, et al. The immunoexpression of heparanase 2 in normal epithelium, intraepithelial, and invasive squamous neoplasia of the cervix. *J Low Genit Tract Dis* 2012;16:256-62.

35. Song L, Gao Y, Wang Z, et al. Clinical changes in serum intercellular adhesion molecule 1 in cervical cancer patients receiving radiotherapy. *Jpn J Radiol* 2024.

36. Ahn KC, Choi JY, Kim JS, et al. Icam-3 endows anticancer drug resistance against microtubule-damaging agents via activation of the icam-3-akt/erk-creb-2 pathway and blockage of apoptosis. *Biochem Biophys Res Commun* 2013;441:507-13.

37. Maruya SI, Myers JN, Weber RS, et al. Icam-5 (telencephalin) gene expression in head and neck squamous carcinoma tumorigenesis and perineural invasion! *Oral Oncol* 2005;41:580-8.

38. Li Y, Gao X, Yang C, et al. Circrna hsa_circ_0018289 exerts an oncogenic role in cervical cancer progression through mir-1294/icmt axis. *J Clin Lab Anal* 2022;36:e24348.

39. Guzman VB, Silva ID, Brenna SM, et al. High levels of granzyme b expression in invasive cervical carcinoma correlates to poor response to treatment. *Cancer Invest* 2008;26:499-503.

40. Gronhoj C, Jensen DH, Agander T, et al. Deep sequencing of human papillomavirus positive loco-regionally advanced oropharyngeal squamous cell carcinomas reveals novel mutational signature. *BMC Cancer* 2018;18:640.

41. Zhai Y, Liu Y, Wang Z, et al. Long non-coding rna linc00313 accelerates cervical carcinoma progression by mir-4677-3p/cdk6 axis. *Onco Targets Ther* 2021;14:2213-2226.

42. Ma Z, Cai Y, Zhang L, et al. Linc00319 promotes cervical cancer progression via targeting mir-147a/igf1r pathway. *Cancer Biother Radiopharm* 2020.

43. Liu H, Zhang L, Ding X, et al. Linc00861 inhibits the progression of cervical cancer&nbsp;cells by functioning as a cerna for mir‑513b‑5p and&nbsp;regulating the pten/akt/mtor signaling pathway. *Mol Med Rep* 2021;23.

44. Liu L, Ren M, Han S, et al. Expression level and clinical significance of low-density lipoprotein receptor-related protein 1b gene in cervical squamous cell carcinoma. *Int J Clin Exp Pathol* 2018;11:1701-1706.

45. Wang X, Jin Y, Xu L, et al. Integrating single-cell rna-seq and bulk rna-seq to construct a novel gammadeltat cell-related prognostic signature for human papillomavirus-infected cervical cancer. *Cancer Control* 2024;31:10732748241274228.

46. Park H, Park SB. Label-free target identification reveals oxidative DNA damage as the mechanism of a selective cytotoxic agent. *Chem Sci* 2019;10:3449-3458.

47. Mai L, Yi F, Gou X, et al. The overexpression of mcph1 inhibits cell growth through regulating cell cycle-related proteins and activating cytochrome c-caspase 3 signaling in cervical cancer. *Mol Cell Biochem* 2014;392:95-107.

48. Wang C, Bai R, Liu Y, et al. Multi-region sequencing depicts intratumor heterogeneity and clonal evolution in cervical cancer. *Med Oncol* 2023;40:78.

49. Honegger A, Schilling D, Bastian S, et al. Dependence of intracellular and exosomal micrornas on viral e6/e7 oncogene expression in hpv-positive tumor cells. *PLoS Pathog* 2015;11:e1004712.

50. Zhang L, Lai Y, Sun Y, et al. Hpv16 e6 regulates the proliferation, invasion, and apoptosis of cervical cancer cells by downregulating mir-504. *Transl Cancer Res* 2020;9:7588-7595.

51. Zou X, Zhu C, Zhang L, et al. Microrna-708 suppresses cell proliferation and enhances chemosensitivity of cervical cancer cells to cddp by negatively targeting timeless. *Onco Targets Ther* 2020;13:225-235.

52. Sharma S, Hussain S, Soni K, et al. Novel microrna signatures in hpv-mediated cervical carcinogenesis in indian women. *Tumour Biol* 2016;37:4585-95.

53. Halle MK, Sundaresan A, Zhang J, et al. Genomic alterations associated with mutational signatures, DNA damage repair and chromatin remodeling pathways in cervical carcinoma. *NPJ Genom Med* 2021;6:82.

54. Yu T, Wu F, Jia Y, et al. Rna n(6)-methyladenosine modification mediates downregulation of nr4a1 to facilitate malignancy of cervical cancer. *Cell Biosci* 2022;12:207.

55. De Marco F, Bucaj E, Foppoli C, et al. Oxidative stress in hpv-driven viral carcinogenesis: Redox proteomics analysis of hpv-16 dysplastic and neoplastic tissues. *PLoS One* 2012;7:e34366.

56. Yang L, Li J, Zang G, et al. Pin1/yap pathway mediates matrix stiffness-induced epithelial-mesenchymal transition driving cervical cancer metastasis via a non-hippo mechanism. *Bioeng Transl Med* 2023;8:e10375.

57. Li S, Gao K, Yao D. Comprehensive analysis of autophagy associated genes and immune infiltrates in cervical cancer. *Iran J Basic Med Sci* 2024;27:813-824.

58. Zhang F, Suarez G, Sha J, et al. Phospholipase a2-activating protein (plaa) enhances cisplatin-induced apoptosis in hela cells. *Cell Signal* 2009;21:1085-99.

59. Ding Y, Wu X, Yang X. Identification of mirnas and target genes associated with lymph node metastasis in cervical cancer using bioinformatics analysis. *Toxicol Mech Methods* 2023;33:625-635.

60. Chen H, Wang J, Yang H, et al. Association between foxm1 and hedgehog signaling pathway in human cervical carcinoma by tissue microarray analysis. *Oncol Lett* 2016;12:2664-2673.

61. Choi YW, Bae SM, Kim YW, et al. Gene expression profiles in squamous cell cervical carcinoma using array-based comparative genomic hybridization analysis. *Int J Gynecol Cancer* 2007;17:687-96.

62. Walline HM, Goudsmit CM, McHugh JB, et al. Integration of high-risk human papillomavirus into cellular cancer-related genes in head and neck cancer cell lines. *Head Neck* 2017;39:840-852.

63. Yang-Chun F, Sen-Yu W, Yuan Z, et al. Genome-wide profiling of human papillomavirus DNA integration into human genome and its influence on pd-l1 expression in chinese uygur cervical cancer women. *J Immunol Res* 2020;2020:6284960.

64. Takayanagi D, Hirose S, Kuno I, et al. Comparative analysis of genetic alterations, hpv-status, and pd-l1 expression in neuroendocrine carcinomas of the cervix. *Cancers (Basel)* 2021;13.

65. Iden M, Tsaih SW, Huang YW, et al. Multi-omics mapping of human papillomavirus integration sites illuminates novel cervical cancer target genes. *Br J Cancer* 2021;125:1408-1419.

66. Su Y, Zhang Y, Zhou M, et al. Genetic alterations in juvenile cervical clear cell adenocarcinoma unrelated to human papillomavirus. *Front Med (Lausanne)* 2023;10:1211888.

67. Shi Y, Liu M, Huang Y, et al. Promotion of cell autophagy and apoptosis in cervical cancer by inhibition of long noncoding rna linc00511 via transcription factor rxra-regulated pld1. *J Cell Physiol* 2020;235:6592-6604.

68. Lopez-Charcas O, Espinosa AM, Alfaro A, et al. The invasiveness of human cervical cancer associated to the function of na(v)1.6 channels is mediated by mmp-2 activity. *Sci Rep* 2018;8:12995.

69. Peng J, Hou F, Zhu W, et al. Lncrna nr2f1-as1 regulates mir-17/sik1 axis to suppress the invasion and migration of cervical squamous cell carcinoma cells. *Reprod Sci* 2020;27:1534-1539.

70. Zhang Y, Wang H, Lu J, et al. Down-regulation of s1pr2 is correlated with poor prognosis and immune infiltrates in cervical squamous cell carcinoma and endocervical adenocarcinoma. *Int J Immunopathol Pharmacol* 2023;37:3946320231178131.

71. Mehterov N, Sacconi A, Pulito C, et al. A novel panel of clinically relevant mirnas signature accurately differentiates oral cancer from normal mucosa. *Front Oncol* 2022;12:1072579.

72. Ohgari Y, Miyata Y, Miyagi T, et al. Roles of porphyrin and iron metabolisms in the delta-aminolevulinic acid (ala)-induced accumulation of protoporphyrin and photodamage of tumor cells. *Photochem Photobiol* 2011;87:1138-45.

73. Qi Y, Yao Q, Li X, et al. Cuproptosis-related gene slc31a1: Prognosis values and potential biological functions in cancer. *Sci Rep* 2023;13:17790.

74. Engelmark MT, Ivansson EL, Magnusson JJ, et al. Polymorphisms in 9q32 and tscot are linked to cervical cancer in affected sib-pairs with high mean age at diagnosis. *Hum Genet* 2008;123:437-43.

75. Kolenda T, Graczyk Z, Zarska B, et al. Sry-related transcription factors in head and neck squamous cell carcinomas: In silico based analysis. *Curr Issues Mol Biol* 2023;45:9431-9449.

76. Xu J, Zou J, Wu L, et al. Transcriptome analysis uncovers the diagnostic value of mir-192-5p/hnf1a-as1/vil1 panel in cervical adenocarcinoma. *Sci Rep* 2020;10:16584.

77. Ma Y, Wang C, Shi M, et al. Searching for the methylation sites involved in human papillomavirus type 16 and 18‑positive women with cervical cancer. *Mol Clin Oncol* 2022;17:149.

78. Beger M, Butz K, Denk C, et al. Expression pattern of ap-2 transcription factors in cervical cancer cells and analysis of their influence on human papillomavirus oncogene transcription. *J Mol Med (Berl)* 2001;79:314-20.

79. Lang X, Huang C, Cui H. Prognosis analysis and validation of fatty acid metabolism-related lncrnas and tumor immune microenvironment in cervical cancer. *J Immunol Res* 2022;2022:4954457.

80. Kawashita S, Matsuda K, Kondo H, et al. Significance of p53-binding protein 1 nuclear foci in cervical squamous intraepithelial lesions: Association with high-risk human papillomavirus infection and p16(ink4a) expression. *Cancer Control* 2020;27:1073274819901170.

81. Zhang X, Wang Y. Identification of hub genes and key pathways associated with the progression of gynecological cancer. *Oncol Lett* 2019;18:6516-6524.

82. Xing X, Tian Y, Jin X. Immune infiltration and a necroptosis-related gene signature for predicting the prognosis of patients with cervical cancer. *Front Genet* 2022;13:1061107.

83. Cavalcanti MS, Schultheis AM, Ho C, et al. Mixed mesonephric adenocarcinoma and high-grade neuroendocrine carcinoma of the uterine cervix: Case description of a previously unreported entity with insights into its molecular pathogenesis. *Int J Gynecol Pathol* 2017;36:76-89.

84. Gu Y, Feng X, Jin Y, et al. Upregulation of mirna-10a-5p promotes tumor progression in cervical cancer by suppressing ube2i signaling. *J Obstet Gynaecol* 2023;43:2171283.
